# High-mass MALDI-MS unravels ligand-mediated G protein–coupling selectivity to GPCRs

**DOI:** 10.1073/pnas.2024146118

**Published:** 2021-07-29

**Authors:** Na Wu, Agnieszka M. Olechwier, Cyrill Brunner, Patricia C. Edwards, Ching-Ju Tsai, Christopher G. Tate, Gebhard F. X. Schertler, Gisbert Schneider, Xavier Deupi, Renato Zenobi, Pikyee Ma

**Affiliations:** ^a^Department of Chemistry and Applied Biosciences, ETH Zürich, CH-8093 Zürich, Switzerland;; ^b^Laboratory of Biomolecular Research, Paul Scherrer Institute, CH-5232 Villigen PSI, Switzerland;; ^c^Department of Biology, ETH Zürich, CH-8093 Zürich, Switzerland;; ^d^Medical Research Council Laboratory of Molecular Biology, Cambridge CB2 0QH, United Kingdom;; ^e^Condensed Matter Theory Group, Paul Scherrer Institute, CH-5232 Villigen PSI, Switzerland

**Keywords:** G protein–coupled receptor, MALDI mass spectrometry, G proteins, coupling selectivity, protein–protein interaction

## Abstract

G protein–coupled receptors (GPCRs) are important pharmaceutical targets for the treatment of a broad spectrum of diseases. Although there are structures of GPCRs in their active conformation with bound ligands and G proteins, the detailed molecular interplay between the receptors and their signaling partners remains challenging to decipher. To address this, we developed a high-sensitivity, high-throughput matrix-assisted laser desorption/ionization mass spectrometry (MALDI-MS) method to interrogate the first stage of signal transduction. GPCR–G protein complex formation is detected as a proxy for the effect of ligands on GPCR conformation and on coupling selectivity. Over 70 ligand–GPCR–partner protein combinations were studied using as little as 1.25 pmol protein per sample. We determined the selectivity profile and binding affinities of three GPCRs (rhodopsin, beta-1 adrenergic receptor [β1AR], and angiotensin II type 1 receptor) to engineered Gα-proteins (mGs, mGo, mGi, and mGq) and nanobody 80 (Nb80). We found that GPCRs in the absence of ligand can bind mGo, and that the role of the G protein C terminus in GPCR recognition is receptor-specific. We exemplified our quantification method using β1AR and demonstrated the allosteric effect of Nb80 binding in assisting displacement of nadolol to isoprenaline. We also quantified complex formation with wild-type heterotrimeric Gα_i_βγ and β-arrestin-1 and showed that carvedilol induces an increase in coupling of β-arrestin-1 and Gα_i_βγ to β1AR. A normalization strategy allows us to quantitatively measure the binding affinities of GPCRs to partner proteins. We anticipate that this methodology will find broad use in screening and characterization of GPCR-targeting drugs.

## Significance

G protein–coupled receptors (GPCRs) are important pharmaceutical targets for the treatment of a broad spectrum of diseases. Upon ligand binding, GPCRs initiate intracellular signaling pathways by interacting with partner proteins. Assays that quantify the interplay between ligand binding and initiation of downstream signaling cascades are critical in the early stages of drug development. We have developed a high-throughput mass spectrometry method to unravel GPCR–protein complex interplay and demonstrated its use with three GPCRs to provide quantitative information about ligand-modulated coupling selectivity. This method provides insights into the molecular details of GPCR interactions and could serve as an approach for discovery of drugs that initiate specific cell-signaling pathways.

G protein–coupled receptors (GPCRs) are the largest family of membrane receptors in humans and play essential roles in physiology and disease (1). Their physiological and cellular signaling effects, modulated by chemically diverse ligands, are exerted through coupling to and activating heterotrimeric G protein complexes (Gαβγ). In humans, there are 16 Gα-subunits that are classified into four families (Gα_s_, Gα_i/o_, Gα_q/11_, and Gα_12/13_). Each Gα-subunit is involved in a specific signal transduction pathway (2). Although our understanding of GPCR signaling has been greatly enhanced by the remarkable progress in GPCR structural biology (3–6), much remains to be discovered to fully understand the molecular mechanisms of allostery and ligand-induced coupling selectivity (or functional selectivity) between GPCRs and their cytoplasmic transducers (G proteins, but also kinases and arrestins) that lead to precise signal transduction cascades and biased signaling (7, 8).

Investigation of the interplay between GPCRs, ligands, and intracellular binding partners is challenging due to the complexity of their interactions. The functional outcome of GPCR activity depends on a still poorly understood network of protein interactions. To date, there are no high-throughput methods to study every G protein and its ability to couple to a given receptor under a standard set of conditions. Many GPCR assays use radio/fluorescence-labeled ligand binding or measurement of second-messenger molecules. More recent methods involve cell-based biosensors, including dynamic mass redistribution and cellular dielectric spectroscopy, that display an overall cellular response and translate GPCR signaling into distinct optical or impedance readouts, respectively (9, 10). However, these assays do not provide a direct readout of G protein coupling to GPCRs. Current biophysical methods that measure such protein interactions directly to provide information on selectivity and affinity—such as surface plasmon resonance, fluorescence resonance energy transfer, isothermal titration calorimetry, and analytical ultracentrifugation—only provide limited information on dynamic protein interactions and either are not suited for high-throughput screening or lack information on all interacting components. Bioluminescence resonance energy transfer (BRET) has been extensively used over the last two decades to study GPCR–protein interactions; however, BRET requires labeling of proteins and, because their level of expression can vary considerably, quantification can be difficult. Native electrospray ionization mass spectrometry (nESI-MS) has been successfully applied to study G protein complexes and membrane proteins (11). However, it is difficult to find buffer conditions that are compatible with both ESI-MS and functional membrane proteins.

Here, we developed a quantitative high-mass matrix-assisted laser desorption/ionization mass spectrometry (MALDI-MS) strategy that combines chemical cross-linking and quantification based on an internal standard to assay the interplay between receptors, ligands, and interacting proteins. Our versatile method enables us to 1) elucidate the selectivity profile of G proteins to GPCRs; 2) dissect the molecular details of complex formation and probe the conformational regulation of GPCRs; and 3) determine binding constant values and characterize ligand–ligand and protein–protein competitions. This method has a much higher tolerance to buffers, salts, detergents, or lipids than ESI-MS (12). Moreover, it does not require any immobilization or chemical labeling of the purified proteins that might alter their bioactivity and the integrity of the complexes during detection. Our high-throughput method (384 sample spots per MALDI plate) is sensitive (the required amount per sample is only 1.25 pmol), rapid (one spectrum can be recorded within 8 s), and quantitative. More than 70 ligand–GPCR–partner combinations were studied.

## Results

### Optimization of the Cross-Linking Reaction and Spotting Method.

The combination of cross-linking and mass spectrometry is a rapidly emerging approach to provide information on the structure and interaction networks of proteins (13, 14). The GPCR–G protein interaction is transient and the complex is considered to be intrinsically unstable (15). Thus, capturing this interaction requires the use of certain stratagems such as stabilization of the complexes with nanobodies or antibodies, or recombinant technology to prevent their dissociation.

**Fig. 1.**
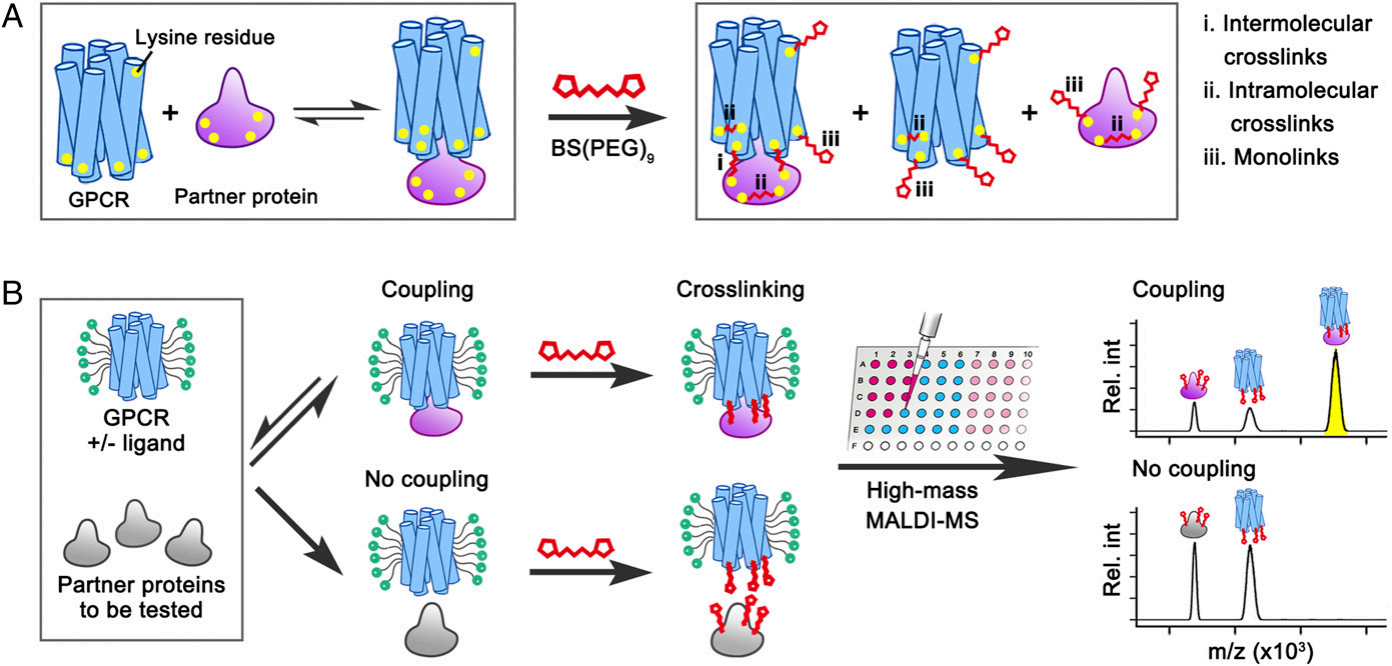
Workflow for the analysis of the selective coupling between GPCRs and partner proteins via high-mass MALDI-MS. (*A*) Schematic of the cross-linking procedure resulting in a stabilized GPCR–G protein complex plus unbound partners “decorated” with monolinks. (*B*) For assessing the ligand-mediated selectivity of a GPCR to a partner protein, the GPCR is first incubated with an mGα-, Nb80, or G protein in the presence or absence of ligand (*SI Appendix*, Table S3). The GPCR–partner complexes formed are then stabilized by chemical cross-linking, followed by detection of the protein components by high-mass MALDI-MS.

Lysine residues are present at the G protein–interacting interfaces of GPCRs (*SI Appendix*, Fig. S1). Based on this, we used PEGylated bis(sulfosuccinimidyl)suberate [BS(PEG)_9_], a bifunctional amine-reactive reagent with a spacer arm length of 38.5 Å (*SI Appendix*, Fig. S1), to cross-link interacting proteins via lysine residues. After reaction, samples will contain intramolecular cross-links, monolinks, and, most importantly, intermolecular cross-links (Fig. 1*A*) that stabilize and capture the protein–protein complexes in their equilibrium state, preventing them from dissociating during the MALDI process. We optimized experimental conditions and cross-linking times using the prototypical photoreceptor rhodopsin (Rho), which couples effectively to mGo (a truncated form of the Gα_o_-subunit) (16) (*SI Appendix*, Fig. S2). We found that even short (≤1-min) preincubation with BS(PEG)_9_ prevents the association between Rho and mGo (*SI Appendix*, Fig. S3), probably due to quick reaction of the cross-linker with lysine residues near the binding interfaces of Rho and mGo, precluding assembly of the complex. Using an optimized experimental procedure, we estimated that in all of the Gα-proteins or their truncated versions tested, six to nine lysine residues react with BS(PEG)_9_ (*SI Appendix*, Table S1), resulting in the formation of approximately two intermolecular cross-links in each complex (*SI Appendix*, Table S2).

GPCRs are extremely challenging integral membrane proteins to work with as they are unstable in detergent solution and require the use of an appropriate condition for their extraction from membranes. Since they are available in low quantity only, a sensitive detection method will therefore help reduce protein sample consumption. Thus, we optimized the MALDI sandwich spotting method by trial and error by testing various chemicals and the number of layers in the sandwiching method, and found that addition of a third layer of saturated sinapinic acid considerably improved the signal level of GPCR proteins by MALDI detection and thus improved sensitivity (*SI Appendix*, *Materials and Methods*). With this sensitivity, we were able to even detect picomole quantities of protein.

### Ligand-Mediated GPCR Selective Coupling.

**Fig. 2.**
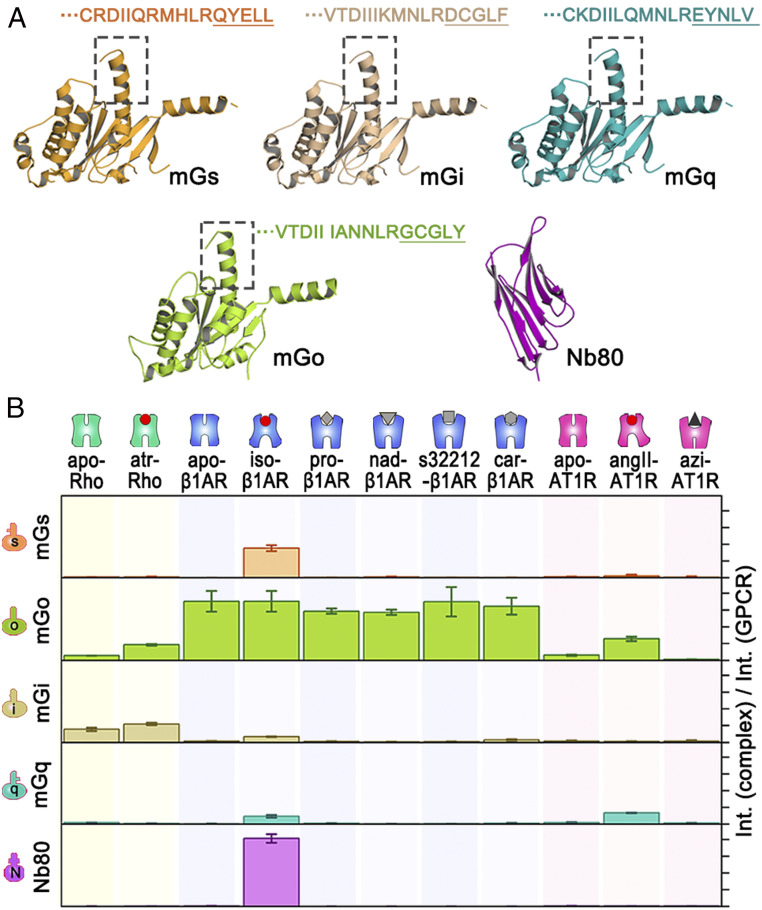
Selectivity in complex formation of apo and ligand-bound GPCRs with partner proteins assayed by high-mass MALDI-MS. (*A*) Three-dimensional structural models of mGα-proteins and Nb80. The amino acid sequences of the C-terminal tail (helix 5; boxes) of the Gα-subunit, accounting for ∼70% of the interacting surface between GPCRs and G proteins, are shown for all mGα-proteins (homology models of mGi and mGq were built using SWISS-MODEL with mGs, Protein Data Bank [PDB] ID code 3SN6, as template); the last five key amino acids in mGα involved in the selectivity determinant are underlined. (*B*) Complex formation propensity of three GPCRs—Rho, β1AR, and AT1R—in the presence or absence of agonists, antagonists, or inverse agonists with their partner proteins mGs, mGo, mGi, mGq, and Nb80 is measured by comparing the relative peak intensity of the GPCR–partner protein complex with that of the noncomplexed GPCR. The ligands used were as follows: angII, angiotensin II; atr, all transretinal; azi, azilsartan; car, carvedilol; iso, isoprenaline; nad, nadolol; pro, propranolol (*SI Appendix*, Table S3); apo designates the ligand-free forms. Error bars represent SDs determined from three independent replicates.

Using our optimized cross-linking protocol, we first showcase our method by examining the coupling ability of three class A GPCRs to a panel of mini–Gα-proteins (17) (hereafter abbreviated as mGα: mGs, mGo, mGi, and mGq) and nanobody 80 (Nb80) (18) in the presence or absence of various ligands (Fig. 2 and *SI Appendix*, Table S3). The GPCRs studied were a constitutively active mutant of bovine Rho, thermostabilized turkey beta-1 adrenergic receptor (β1AR), and the F117W mutant of mouse angiotensin II type 1 receptor (AT1R) (for protein sequences, see *SI Appendix*, Table S4).

Detection and analysis of multicomponent protein complexes (such as GPCRs with their heterotrimeric G proteins) by any biophysical method are challenging. We therefore established our method by using mGα-proteins, which are simplified versions of their full-length counterparts (Gα) containing the GTPase domain but lacking the α-helical domain, and are widely used in biochemical, biophysical, cellular, and structural biology studies for studying GPCR–G protein interactions and GPCR activation mechanisms (6, 11, 19, 20). Swapping the C tail (α5-helix) of the G protein is commonly performed to switch selectivity between G protein subtypes (21). Our mGo and mGs are thermostabilized versions of their truncated wild-type G proteins, and mGq and mGi are engineered from mGs by introducing nine and seven mutations on the α5-helix that correspond to residues of Gq and Gi, respectively (17). Mixing and incubation of the binding partners are followed by treatment with BS(PEG)_9_, and the resulting complexes and remaining unbound partners in the sample are detected by high-mass MALDI-MS by monitoring the peak intensities of each species. Examples of measured spectra are shown in Fig. 1*B*, the results are summarized in Fig. 2, and the full dataset for all combinations is shown in *SI Appendix*, Fig. S4. Our method allows us to detect conformational changes and ensembles of the receptor by following receptor–complex formation, which can be read out directly from the mass spectra.

GPCR orthosteric ligands fall into three categories: activating (agonists), inactivating (inverse agonists), and neutral (antagonists). Our assay largely displays the expected GPCR–G protein recognition patterns. The constitutively active Rho mutant couples to the two members of the Gα_i/o_-family, mGo and mGi, both in the apo (apo-Rho) and agonist-bound (atr-Rho) forms (Fig. 2). This was expected, as constitutively active Rho has been shown to strongly recruit Gi and Go (16, 22, 23). The iso-β1AR was found to bind to Nb80 (a Gs mimetic nanobody), proving that our β1AR construct can achieve a fully active conformation and that Nb80 binding is conformation-specific (24). It has been shown that this receptor can couple to Gα_s_-, Gα_i_-, and Gα_q_-families (25) and, indeed, we observe that agonist-bound β1AR (iso-β1AR) can couple to some extent to all mGα-subtypes (Fig. 2). Apo-β1AR can specifically couple to mGo, which showed similar selectivity profiles with known antagonists (propranolol, nadolol, and carvedilol) and s32212. Based on these profiles, we can classify s32212 as an antagonist for β1AR. Finally, we observed that our agonist-bound AT1R (angII-AT1R) couples to both mGq and mGo, but not mGi (Fig. 2). This could be because our mGi construct lacks some key residues required for receptor binding (17). As mGi is engineered from mGs and contains only the Gi fragment on the α5-helix, this suggests the α5-helix of Gi is not the main determinant for its coupling to AT1R and instead the globular part of Gi could be more important. This may also explain why we observe a weak interaction of mGi with iso-β1AR and potentially weak interactions also with car-β1AR and angII-AT1R (Fig. 2). Azilsartan, a potent inverse agonist, can displace many AT1R blockers from the receptor (26). We expect that this ligand stabilizes the receptor in an inactive conformation with severely impaired mGα-coupling. Indeed, this ligand abolished coupling of all mGα-proteins to AT1R, including mGo (Fig. 2). These data illustrate how the apo, agonist-bound, antagonist-bound, and inverse agonist-bound forms of receptors exist in different conformational ensembles with different profiles of G protein recognition.

From the perspective of the mGα-proteins, mGo is found to be the most promiscuous G protein, as it binds to all agonist/antagonist-bound receptors and, remarkably, to all apo receptors (Fig. 2 and *SI Appendix*, Fig. S4). Native Go protein is highly expressed in the central and peripheral nervous systems, endocrine cells, and cardiomyocytes, being the most abundant G protein subtype in neurons (27, 28). There is considerable evidence for the existence of functional complexes of apo-GPCRs with G protein (29–33) and the Go subtype seems particularly predisposed to such precoupling (34, 35). Thus, we conjecture that the promiscuity of mGo observed in our assay represents its ability to recognize apo (through precoupling), agonist-bound, and antagonist-bound receptors.

### A Normalization Strategy to Determine the Binding Affinity of GPCR–Partner Complexes.

**Fig. 3.**
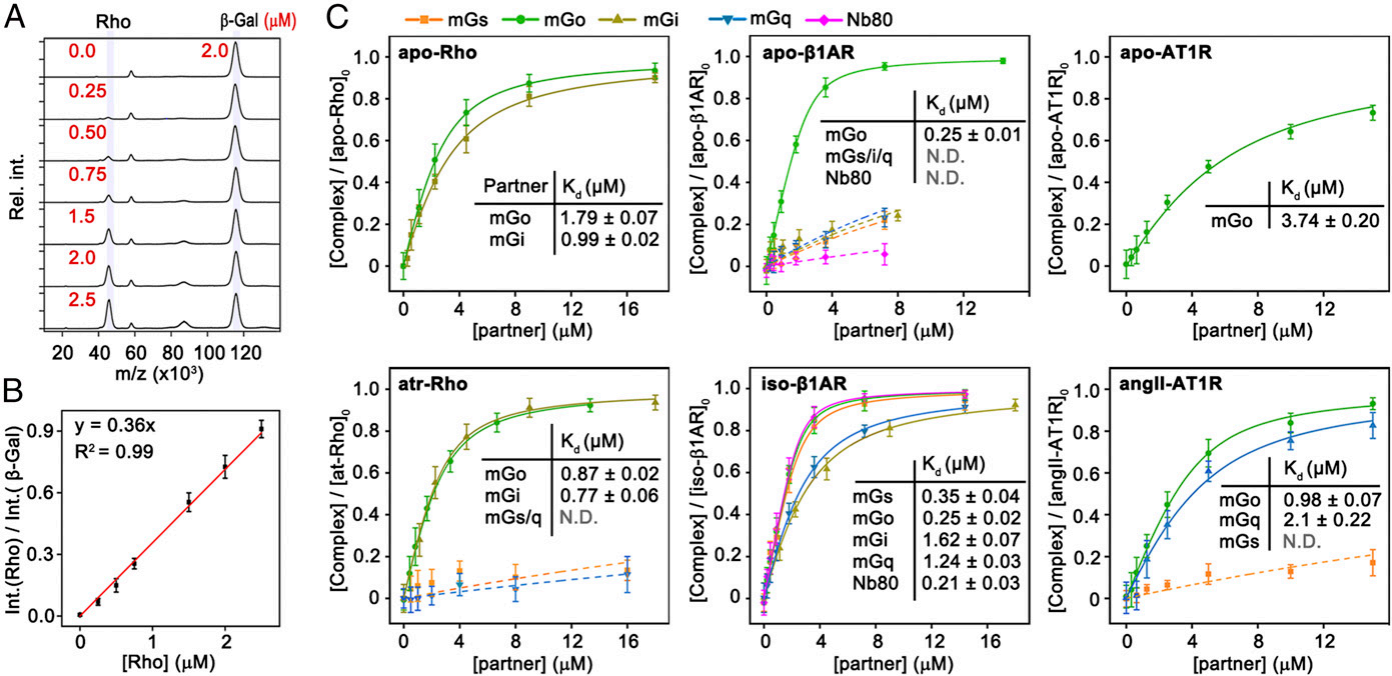
Binding affinities between GPCRs and partner proteins. (*A*) Calibration of different concentrations of Rho normalized to 2 µM β-galactosidase. (*B*) Peak intensity ratio of Rho to β-galactosidase vs. Rho concentration in the sample. (*C*) Evaluation of the affinities (dissociation constants; µM) for different GPCRs with various partner proteins (mGs, orange; mGo, green; mGi, beige; mGq, turquoise; Nb80, magenta), using both apo (*Top*) and ligand-bound (*Bottom*) forms of the GPCRs. The data were obtained by titrating the G protein against the GPCR in 20 mM Hepes buffer (pH 7.5), 40 mM NaCl, 0.01% lauryl maltose neopentyl glycol. Error bars represent SDs from three independent replicates. N.D., not determined.

Since ionization efficiencies of proteins are highly variable in MALDI and could change upon cross-linking, there is no direct correlation between peak intensity and protein concentration. To be able to quantify individual protein components in the spectra, we developed a normalization strategy using β-galactosidase (β-gal) as a reference protein (examples of calibration and a standard curve for Rho are shown in Fig. 3 *A* and *B*, and the rest of the data are shown in *SI Appendix*, Fig. S5), which is stable in its monomeric form (*SI Appendix*, Fig. S6) and does not interfere with the analytes of the sample (*SI Appendix*, Figs. S7 and S8). This allowed us to calculate the concentrations of each species at equilibrium (*SI Appendix*, Figs. S9–S11) and the corresponding dissociation constants (*K*_d_s) of the complexes between GPCRs and their partner proteins (Fig. 3*C*).

The measured *K*_d_s between the GPCRs and interacting proteins are in the high nanomolar–to–low micromolar range (summarized in Fig. 3 and *SI Appendix*, Table S5). Literature *K*_d_ values are scarce because such measurements are challenging. A comparison of the MALDI-based *K*_d_ data with the literature and a microscale thermophoresis measurement showed good agreement (*SI Appendix*, Fig. S12 and Table S6). We observed that mGo generally had a higher affinity to the GPCRs compared with other partner proteins (Fig. 3). For β1AR, the *K*_d_ of mGo (0.25 μM) was hardly influenced by the ligands (Fig. 2 and *SI Appendix*, Fig. S4) and was considerably lower than that of mGs (0.35 μM), mGq (1.24 μM), and mGi (1.62 μM). Among the receptors, β1AR generally has higher affinities to the test partner proteins. For AT1R, binding to mGo is twice as strong as to mGq (Fig. 3 and *SI Appendix*, Fig. S4 and Table S5). We quantitatively elucidated the interaction strength between the protein–protein complexes. These interactions are the key determinant of information transmission within a signaling network.

### Effect of the G Protein C Terminus on the Interaction with GPCRs.

**Fig. 4.**
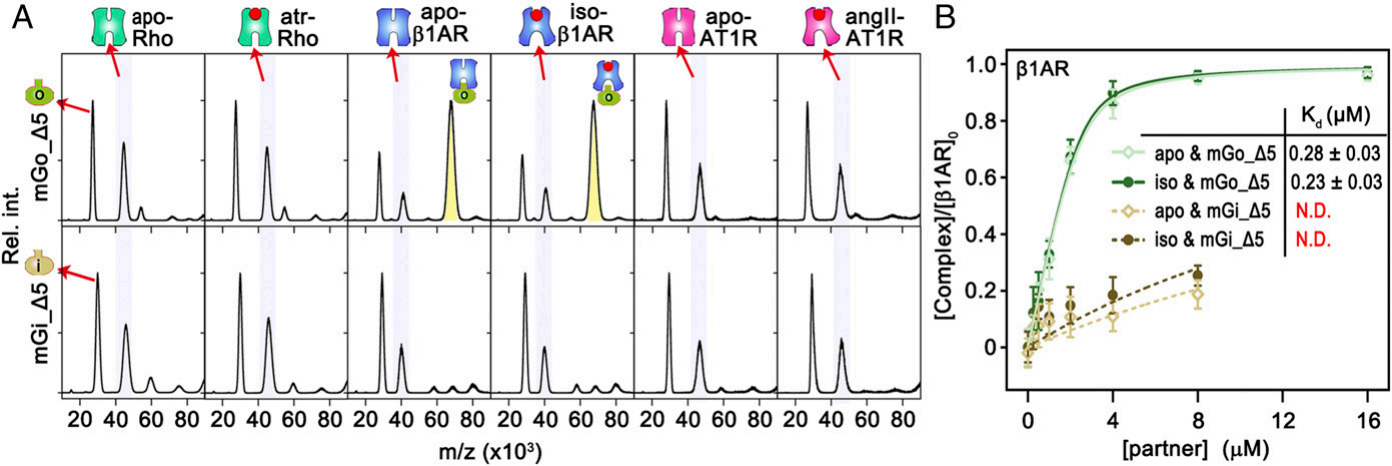
Role of the C terminus of mGo and mGi in binding to GPCRs. (*A*) Mass spectra showing the coupling between ligand-bound GPCRs (from *Left* to *Right*: apo-Rho, atr-Rho, apo-β1AR, iso-β1A, apo-AT1R, angII-AT1R) and truncated mGo (mGo_∆5; first row) and mGi (mGi_∆5; second row) proteins. (*B*) *K*_d_ values of apo-β1AR–mGo_∆5 (light green empty squares), iso-β1AR–mGo_∆5 (dark green solid circles), apo-β1AR–mGi_∆5 (light brown empty squares), and iso-β1AR–mGi_∆5 (dark brown solid circles). Error bars represent SDs from three independent repeats.

Many aspects of the formation of signaling complexes between GPCRs and G proteins are still unclear, such as the molecular determinants of coupling selectivity (8) or the role of precoupling of G proteins to inactive receptors (34). Recent structural and biophysical studies have confirmed the C terminus of the Gα-subunit as one of the primary determinants of the interaction with GPCRs (36, 37). The binding characteristics of our mGα-constructs show indeed that a few amino acid substitutions in the C terminus of mGs, mGi, and mGq can alter their selective coupling to AT1R and Rho and impact the binding affinity to β1AR (Fig. 3). To further assess the role of the mGα–C terminus, we truncated the last five residues from mGo and mGi (mGo_Δ5 and mGi_Δ5) and assessed their binding affinity to our panel of receptors. Our data show that mGi truncation abolished coupling to both apo and agonist-bound receptors (Fig. 4 and *SI Appendix*, Fig. S13). However, truncation of mGo affected coupling to Rho and AT1R, but not to β1AR, which still bound mGo_Δ5 with similar affinities to mGo in both the apo (0.28 μM) and agonist-bound (0.23 μM) states. This indicates that the last five residues of G protein are not always the main determinant for receptor recognition and other regions can mediate high-affinity binding (15, 21). Based on the observation that ligands did not affect the affinity between β1AR and mGo but had a significant effect on the binding of Rho and AT1R to mGo, we speculate that ligand-induced GPCR conformational changes have a greater influence on the C-terminal contribution of the binding to G protein, and that GPCR and mGo interactions are receptor-dependent.

### Ligand-Mediated Competition between Partner Proteins.

**Fig. 5.**
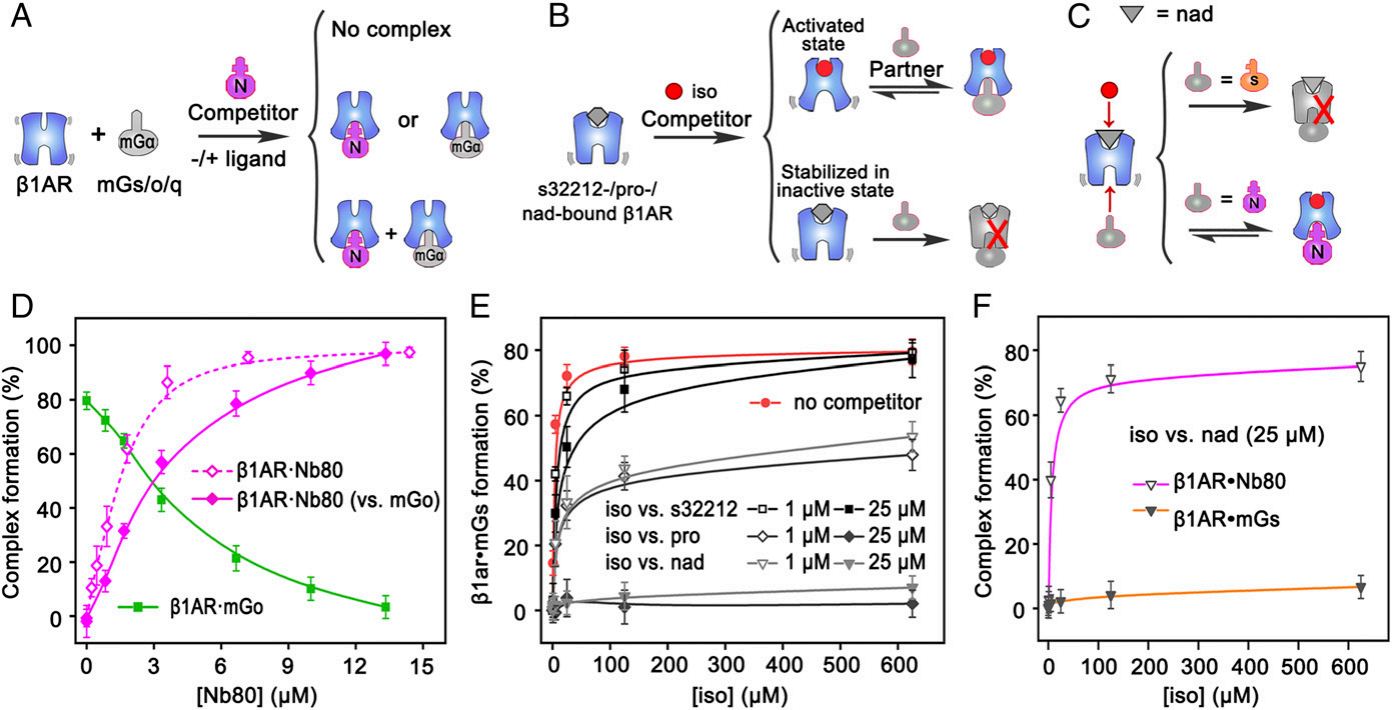
Competition between partner proteins and between ligands for binding to GPCRs. (*A*) Schematic of the competition between Nb80 and other mGα proteins (mGs, mGo, and mGq) for binding to β1AR (in the presence or absence of ligand) and the different assembly possibilities. (*B*) Schematic of GPCR conformational ensembles induced by the competition between antagonist and agonist ligands. The GPCRs are stabilized in a suitable conformation under the combined effect of both ligands and partner proteins. (*C*) Schematic of the competition between nadolol and isoprenaline and the formation of the β1AR–Nb80 complex, modulated by the presence of a partner protein. (*D*) Conversion of β1AR–mGo (solid green squares) to β1AR–Nb80 (solid magenta diamonds) using 2.5 μM β1AR, 3.0 μM mGo, and increasing concentrations of Nb80, and conversion to β1AR–Nb80 in the absence of mGo (empty magenta diamonds). (*E*) β1AR–mGs complex formation modulated by different ligands at different concentrations of isoprenaline. (*F*) Comparison of β1AR–mGs and β1AR–Nb80 complex formation as revealed by titration with isoprenaline. Error bars represent SDs from three independent repeats.

To explore the interplay between affinity and selectivity in GPCR-binding partners, we measured the formation of β1AR complexes with mGα-proteins (mGs, mGo, and mGq) in the presence of the competitor Nb80 at equimolar amounts (Fig. 5*A* and *SI Appendix*, Fig. S14 *A* and *B*). In the absence of ligand, β1AR binds only to mGo due to its precoupling ability (*K*_d_ of 0.25 µM) (Fig. 3), indicating that the ligand-free receptor ensemble is conformationally specific for mGo only. Isoprenaline-bound β1AR selectively coupled with Nb80 in the presence of mGs or mGq, but coupled with both mGo and Nb80. This is due to the tighter binding of Nb80 for isoprenaline-bound β1AR (0.21 µM) compared with mGs (0.35 µM) and mGq (1.24 µM), while mGo binds with similar affinity to Nb80 (0.25 µM) (Fig. 3).

To measure the inhibition ability of Nb80 to mGo, we measured the formation of β1AR–mGo complexes at increasing concentrations of Nb80 (Fig. 5*D* and *SI Appendix*, Fig. S14*C*), and calculated the inhibitory constant (*K*_i_) of Nb80 to mGo (1.57 ± 0.24 μM) (*SI Appendix*, Fig. S14 *D* and *E*). We also measured the effects of isoprenaline on the competition between mGo and Nb80 and, as expected, the competitiveness of Nb80 increased with rising isoprenaline concentration (*SI Appendix*, Fig. S15). These results show that when multiple partner proteins coexist, while GPCRs prefer to couple with partners of higher affinity, changes in ligand and partner concentrations can alter this coupling selectivity. We can substantiate that the promiscuous binding of mGo is specific for the two following reasons: First, we were able to displace mGo binding to AT1R in the presence of the inverse agonist azilsartan, showing that mGo binding can be allosterically modulated by ligands (Fig. 2*B*). Second, Nb80 can also displace mGo binding to β1AR in a competitive manner (Fig. 5*D*). These results strongly suggest that mGo binds to the “canonical” recognition site on the cytoplasmic side of the activated receptor.

### Allosteric Influence of Ligands on GPCRs.

We also investigated the allosteric conformational regulation of GPCR–G protein complexes by several ligands (Fig. 5 *B* and *C* and *SI Appendix*, Fig. S16). All antagonists tested had the same effect on the coupling ability of β1AR, which binds only to mGo in their presence (Fig. 2). To further characterize these antagonists, we measured their ability to compete with the agonist and affect formation of the receptor–mGα complexes by incubating 2.5 µM apo-β1AR with equimolar amounts (50 µM) of antagonist (s32212, propranolol, carvedilol, or nadolol) and agonist (isoprenaline) (Fig. 5 *B* and *C* and *SI Appendix*, Fig. S16). At these concentrations, isoprenaline cannot displace propranolol or carvedilol from the receptor, and propranolol/carvedilol-bound β1AR still only recruits mGo, but it can displace s32212 and recovers coupling to mGs, Nb80, and, partially, mGq. Interestingly, in nadolol-bound β1AR, isoprenaline only partially recovers its recruiting ability with Nb80, but not with mGs and mGq (*SI Appendix*, Fig. S16).

We next explored in more detail the inhibitory ability of these antagonists on the formation of GPCR complexes. For that, we measured the formation of the β1AR–mGs and β1AR–Nb80 complexes in the presence of 1 or 25 µM antagonists at increasing concentrations of isoprenaline (Fig. 5 *E* and *F* and *SI Appendix*, Fig. S17). s32212 behaves as a surmountable competitive antagonist, as raising the isoprenaline concentration recovers near-maximal formation of the β1AR–mGs complex (80%); the *K*_i_ of s32212 was determined to be 3.56 ± 0.26 µM (*SI Appendix*, Figs. S17 and S18). On the contrary, propranolol behaves as an insurmountable competitive antagonist, as isoprenaline (at any concentration) cannot recover maximal β1AR–mGs complex formation. Nadolol shows dual behavior in different complex systems: It is insurmountable in β1AR–mGs but surmountable in β1AR–Nb80 (Fig. 5*F*), likely due to the higher affinity of Nb80 to isoprenaline-bound β1AR compared with mGs, and the allosteric effect of Nb80, which assists displacement of nadolol to isoprenaline. The positive cooperative effect of Nb80 on isoprenaline binding we observe here is consistent with a previous report (38) and demonstrates the allosteric mechanistic property of GPCRs. Our data agree with the concept that ligands induce (or stabilize) specific receptor conformations and the sensitivity of our method reveals in detail the complexity of their interactions. We showed that nadolol is more surmountable than propranolol, in agreement with their reported p*K*_i_ values (−8.2 and −7.2, respectively) (*SI Appendix*, Table S3). Furthermore, we show that s32212 is a weaker antagonist for β1AR than nadolol, as shown by its less prominent inhibitory effect (*SI Appendix*, Fig. S16).

### Ligand-Biased Assembly of the β1AR–G Protein/Arrestin Complexes.

**Fig. 6.**
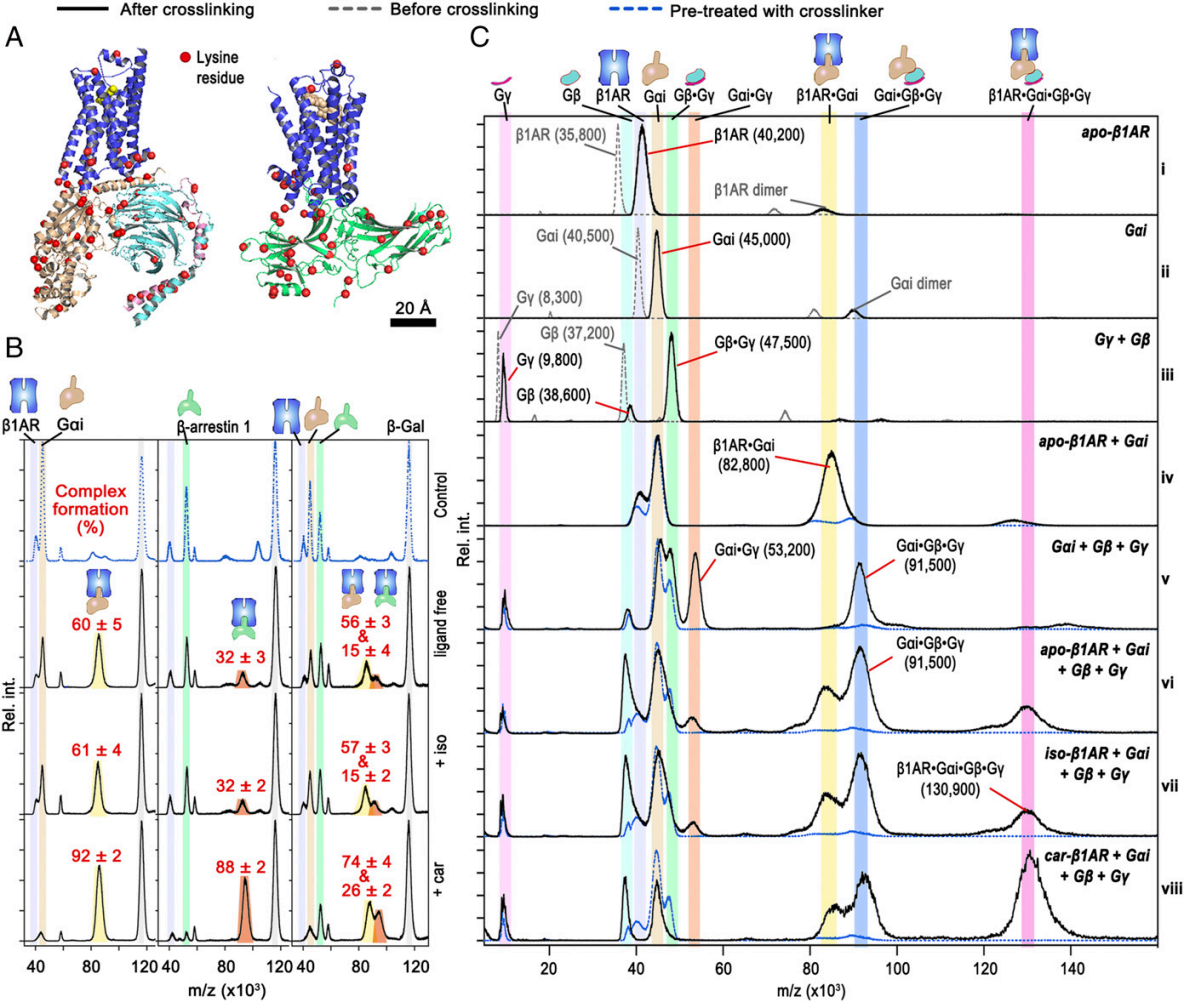
Ligand-biased binding between β1AR and Gi/arrestin proteins. (*A*) Structural models of the pentameric complex β1AR–Gα_i_–Gβ–Gγ with bound isoprenaline (*Left*; assembled using molecular graphics software [PyMOL] and the templates PDB ID codes 3SN6, 2Y03, and 1GP2) and β1AR–β-arrestin-1 complex (*Right*; PDB ID code 6TKO) with lysine residues highlighted in red. (*B*) Control experiment showing the absence of complex formation if the interaction partners are first treated with cross-linker (*Top*), and complex formation between β1AR and Gα_i_/arrestin/Gα_i_ + arrestin in ligand-free and isoprenaline- and carvedilol-bound receptors. Complex formation in percentages was calculated by normalization with β-Gal as a standard. (*C*) Formation of diverse complexes of Gα_i_, β, Gγ, and β1AR following incubation and treatment with BS(PEG)_9_ in the absence and presence of isoprenaline or carvedilol. Gray dashed traces are spectra recorded without applying cross-linker; blue dashed traces are spectra recorded after pretreating mixture components with cross-linker before incubation. Percentage complex formation is calculated from three independent repeats.

Next, we expanded our method by using full-length wild-type protein partners—Gα_i_βγ and β-arrestin-1 (Fig. 6). We first incubated apo or isoprenaline- or carvedilol-bound β1AR with Gα_i_, Gα_i_–Gβ–Gγ, or β-arrestin-1 at equimolar concentration and tested the formation of β1AR–protein complexes. Artifacts were excluded by measuring mixtures of proteins that were pretreated with the cross-linker, which could not form protein complexes (Fig. 6*B*). We found that isoprenaline-bound β1AR and ligand-free β1AR exhibited similar binding affinity to Gα_i_ and arrestin (∼60 and 32% complex formation, respectively), while carvedilol-bound β1AR showed a higher affinity to Gα_i_ and arrestin (∼92 and 88% complex formation, respectively). We also tested the complex formation in an equimolar mixture of β1AR, Gα_i_, and arrestin. We found that both the β1AR–Gα_i_ and β1AR–arrestin complexes were present, but that the former formed much more readily than the latter (four times higher intensity with apo- or iso-β1AR and three times higher intensity with car-β1AR). This also illustrates that Gα_i_ possesses a higher binding affinity to β1AR than arrestin.

We then studied the interaction between ligand-bound β1AR and Gα_i_–Gβ–Gγ. We incubated Gα_i_ with Gβ–Gγ at equimolar concentration and, as expected, we detected peaks for the cross-linked complexes Gβ–Gγ (47,500 Da) and Gα_i_–Gβ–Gγ (91,500 Da) (Fig. 6*C*). Additionally, we observed a peak *m/z* at 53,200 Da corresponding to a cross-linked complex of Gα_i_ with Gγ (Fig. 6*C* and *SI Appendix*, Fig. S19). Following addition of β1AR, we observed the simultaneous presence of the cross-linked complexes Gα_i_–Gγ, Gα_i_–Gβ–Gγ, β1AR–Gα_i_ (82,800 Da), and β1AR–Gα_i_–Gβ–Gγ (130,900 Da) (Fig. 6*C*). The presence of isoprenaline hardly altered the relative intensity of these protein peaks compared with the absence of ligand, while carvedilol increased the formation of β1AR–Gα_i_–Gβ–Gγ, resulting in a complete disappearance of the β1AR, Gβ–Gγ, and Gα_i_–Gγ peaks. As car-β1AR does not bind mGi (Fig. 2), these data show that mGi did not inherit all the bioactivity from Gi, indicating that other regions of the Gα–core domain make a large contribution to its receptor-binding specificity. Our receptors were not treated with kinases or phosphorylation enzymes; in addition, our β1AR construct is truncated at the C terminus and intracellular loop 3, meaning that the majority of the phosphorylation sites are absent. The absence of phosphorylation, which precludes protein kinase A–dependent Gs/Gi switching in β1AR (39), is the probable cause of the lack of Gα_i_–Gβ–Gγ recruitment observed for iso-β1AR (i.e., same response as the apo receptor; Fig. 6*C*). Moreover, our data suggest that carvedilol-mediated arrestin coupling to β1AR is phosphorylation-independent. Importantly, our method allows the quantification of Gi and arrestin complex formation induced by carvedilol, which quantitatively shows how ligands modulate the extent of the recruitment of G proteins and arrestin.

## Discussion

Several recent technological advances have enhanced our understanding of various aspects of GPCR activation mechanisms and signaling. For example, structural biology studies by NMR, X-ray crystallography, and cryo-EM have provided high-resolution structural insights, enabling the molecular characterization of different protein complexes. In addition, functional studies using biophysical and signaling assays have allowed the characterization of ligand properties and ligand-mediated cellular response. However, the characterization of the network of GPCR–protein interactions following receptor activation remains difficult to tackle. While the traditional view of GPCR signaling involves a more or less sequential course of events, it is now clear that receptors can adopt multiple active states and engage multiple intracellular binding partners in a complex interaction network. To better understand the network of ligand-mediated GPCR–G protein interactions, we developed a method to address this by directly monitoring the GPCR–protein complex formation. We demonstrated the use of our method by screening three class A GPCRs against a panel of engineered Gα-proteins and generated a selectivity profile for each ligand tested (Fig. 2*B*). In agreement with a previous study (21), a Gi/o-coupled receptor (Rho in this case) is more selective and couples only to Gi and Go. Our Gs- and Gq-coupled receptors (β1AR and AT1R) are more promiscuous and always couple to some extent to the Gi/o family as well (Fig. 2*B*). In order to fully understand the promiscuity of agonist-bound receptors, probably high-resolution structures of the same receptor bound to different transducers would be required to provide the molecular details and insights into this aspect.

The selectivity profiles of our three GPCRs indicate that each ligand-free or ligand-bound receptor has its unique coupling profile (Fig. 2*B*). Concurring with previous studies, we also show that agonist-bound GPCRs exist in multiple conformations (Fig. 2). This explains the complexity of the GPCR-signaling mechanism, which is not governed simply by “active” and “inactive” states, or a ternary model. The method presented here allows us to quantitatively investigate GPCR interactions. The proportion of different ligands (agonist and antagonist) can further fine-tune the receptor conformational ensembles (*SI Appendix*, Fig. S16). Thus, our data enable us to observe the allosteric conformational regulation of GPCRs, which helps to explicate the plasticity of GPCR signal transduction.

The development and application of efficient GPCR-binding assays are critical in the early stages of drug development. Current high-throughput technologies for assaying the function of GPCRs mainly depend on the measurement of second-messenger output, such as inositol phosphate, calcium, and cyclic adenosine monophosphate. These readouts are distant from the actual information of the GPCR–effector complex, and rely on cellular responses that can be modulated by several separate or even cross-talking signaling pathways. Therefore, the second-messenger output does not directly indicate the “recruiting” activity of a ligand and does not provide an accurate way to profile ligands according to this measure. Unraveling the relationships between ligand, receptor, and the coupling complexes (with G proteins and arrestins) that mediate downstream signaling events is the key to unscramble allosterism and biased signaling. We showed that our method can effectively be used to study the coupling of both G protein and arrestin (Fig. 6) and thus could potentially be used in drug discovery for ligand profiling.

Investigating the pentameric complex system (ligand–β1AR–Gα_i_–Gβ–Gγ) (Fig. 6*C*) was more complicated than the three-component systems (ligand–GPCR–mGα/Gα/arrestin) and posed a challenge to obtain the binding affinity values for all components. However, our data provide a unique profile for such pentameric systems at equilibrium (Fig. 6*C*). Further expansion of our method to study other members of the G protein, arrestin, and G protein kinase families may be of great relevance to future GPCR deorphanization approaches, or to dissect partially overlapping signaling pathways occurring in some of the G protein families, such as the Gi/o/z.

GPCRs are allosterically dynamic proteins. Multiple biophysical techniques are currently being used to fully understand how different ligands produce different signaling patterns. Complementary to previous techniques, our strategy represents a mass spectroscopy method that allows characterization of direct ligand-induced receptor–protein complex formation in detail. We developed a powerful all-in-one method, unraveling the G protein–coupling selectivity to GPCRs and receptor conformational regulation, to provide information regarding protein/analyte concentrations, their competition, affinity constants, molecular size, and structure. We therefore anticipate that our method will emerge as a valuable strategy for high-throughput screening and for unraveling the molecular details of ligand–GPCR–protein interaction.

## Materials and Methods

Detailed materials and methods are provided in *SI Appendix*, *Materials and Methods*. This includes detailed information about materials used, methodology and experiment protocols, mass spectra and data analysis, microscale thermophoresis data, three-dimensional models of the tested proteins, tables of the number of intermolecular cross-links present in each complex, information on ligands, and amino acid sequences of the proteins.

## Supplementary Material

Supplementary File

## Data Availability

The original data used in this publication have been made available in a curated data archive at Eidgenössche Technische Hochschule Zürich (https://www.researchcollection.ethz.ch) under the DOI 10.3929/ethz-b-000495712 (40)
