# Supplementary material for: High-mass MALDI-MS unravels ligand-mediated G protein–coupling selectivity to GPCRs
Source: Proc Natl Acad Sci U S A. 2021 Jul 29;118(31):e2024146118. doi: 10.1073/pnas.2024146118 (PMC8346855; doi:10.1073/pnas.2024146118)
Supplement: Supplementary File [file pnas.2024146118.sapp.pdf]

PNAS
www.pnas.org

# Supplementary Information for

High-mass MALDI-MS unravels ligand-mediated G-protein coupling selectivity to GPCRs

Na Wu[a], Agnieszka M. Olechwier[b,c], Cyrill Brunner[a], Patricia C. Edwards[d], Ching-Ju Tsai[b], Christopher G. Tate[d], Gebhard F.X. Schertler[b,c], Gisbert Schneider[a], Xavier Deupi[b,e], Renato Zenobi[a]*, Pikyee Ma[b*]

*Correspondence to: Renato Zenobi (zenobi@org.chem.ethz.ch); Pikyee Ma (pik-yee.ma@psi.ch)

**This PDF file includes:**

SI Materials and Methods
Figures S1 to S19
Tables S1 to S6
SI References

## SI Materials and Methods

**Chemicals**. PEGylated bis (sulfosuccinimidyl) suberate ($BS(PEG)_9$), $BS(PEG)_5$, and disuccinimidyl suberate (DSS) were purchased from Thermo Fisher Scientific (USA). β-Galactosidase (β-Gal), isoprenaline hydrochloride (iso), azilsartan (azi), angiotensin II (Ang II, human, >=93%), all trans-retinal (atr), (S)-(-)-propranolol hydrochloride (pro), nadolol (nad), tris(2-carboxyethyl) phosphine (TCEP), carvedilol (car), glycerol, 1D4 peptide, dithiothreitol (DTT), protease inhibitors, DNAse, lysozyme, imidazole, TEV protease, Terrific Broth (TB) media, glucose, isopropyl β-d-1-thiogalactopyranoside (IPTG), sucrose, and guanosine 5'-diphosphate sodium salt (GDP) were purchased from Sigma-Aldrich. Sinapinic acid (SA) was purchased from Tokyo Chemical Industry (Eschborn). Lauryl maltose neopentyl Glycol (LMNG) and cholesteryl hemisuccinate (CHS) were purchased from Anatrace. S32212 was purchased from Tocris. Hepes (1.0M buffer solution, pH 7.5) was purchased from ABCR GmbH. 2nd generation RED-maleimide dye was purchased from NanoTemper Technologies (Munich).

**Expression and purification of AT1R**. Mouse AT1R with F117W Δ324 and a C-terminal 1D4 tag was cloned into pcDNA4/TO and stably transfected into HEK293 GntI cells (1) containing a stably integrated repressor pcDNA/TR. Cells were grown to a density of 3-4 x $10^6$ cells/mL, induced with tetracycline and sodium butyrate, and harvested 48-72 h post-induction. Through extensive screening, we identified conditions for stabilising the receptor in the apo-state where no ligands (agonist or antagonist) were added in any purification steps. Cells were lysed in hypotonic solution comprised of 10 mM Hepes pH 7.5, 2 mM EDTA, 10 mM $MgCl_2$ and Roche protease inhibitors cocktail. Cell membranes prepared were solubilised with LMNG for 1.5 h, followed by

removal of insoluble fraction by centrifugation at 35,000 rpm for 30 min. The supernatant was bound to 1D4 resin (cyanogen bromide-activated Sepharose 4B resin from GE Healthcare Life Science, Freiburg, Germany, bound with anti-1D4 antibody from Cell Essentials, Boston, MA) overnight. The next day, the resin was washed with wash buffer (20 mM Hepes pH 7.5, 300 mM NaCl, 0.01% LMNG and 0.001% CHS and 10 mM $MgCl_2$) and 1D4 tagged-AT1R was eluted with 20 mM Hepes pH 7.5, 100 mM NaCl, 0.01% LMNG, 0.001% CHS, 10 mM $MgCl_2$, 320 µM 1D4 peptide and 10% glycerol. Eluted AT1R was concentrated and subjected to size exclusion chromatography (SEC) on Superdex 200 Increase column (GE) equilibrated in buffer 20 mM Hepes pH 7.5, 100 mM NaCl, 0.01% LMNG, 0.001% CHS and 10% glycerol. Peak fractions corresponding to the pure AT1R protein were pooled together, flash frozen in liquid nitrogen and stored at -80$^{o}$C.

**Expression and purification of β1AR**. Expression and purification of turkey apo-β1AR were performed as described previously (2), but with some modifications. Turkey β1AR was cloned into pcDNA4/TO vector and expressed in stable HEK293 GntI- cells (1). Cells were grown to density of 3-4 x $10^6$ cells/mL, induced with tetracycline and sodium butyrate, and harvested 48-72 h post-induction. Detergent screening was performed to optimization the solubilization step for apo-protein. Membranes were prepared and solubilized for 1 hour in LMNG detergent, followed by removal of insoluble fraction by centrifugation at 40,000 rpm for 1.5 hours. Solubilised fraction was bound to 1D4 resin for 3 hours. The resin was washed with 20 mM Hepes pH 7.5, 300 mM NaCl, 0.01% LMNG and 0.001% CHS, and β1AR was eluted through cleavage buffer with HRV3C protease containing 20 mM Hepes 7.5, 100 mM NaCl, 0.01% LMNG and 0.001% CHS.

Eluted β1AR protein was concentrated and subject to SEC on Superdex 200 Increase column (GE) equilibrated in 20 mM Hepes pH 7.5, 100 mM NaCl, 0.01% LMNG and 0.001% CHS. Pure β1AR protein in the peak fractions was collected, concentrated, flash frozen in liquid nitrogen and stored at -80°C.

**Expression and purification of Rhodopsin**. Preparation of a constitutively active bovine rhodopsin N2C/M257Y/D282C mutant was prepared as described previously with minor modifications (3). During the immunoaffinity purification using 1D4 antibody, the apo protein was reconstituted with all-trans retinal. Excess retinal was washed off with 8 column volume (CV) of PBS buffer containing 0.04% DDM and 25 CV of buffer containing 20 mM Hepes (pH 7.5), 150 mM NaCl, 0.005% LMNG, 0.0005 % CHS. Rhodopsin was eluted three times with 1.5 CV of buffer containing 20 mM Hepes pH 7.5, 150 mM NaCl, 1 mM $MgCl_2$, 10% glycerol, 80 μM 1D4 peptide, 0.005% LMNG, 0.0005% CHS. Eluted fractions were combined and concentrated using a VIVASPIN 20 concentrator [molecular weight cut-off (MWCO), 100,000] (Satorius) to 2.72 mg/ml. Finally, the protein was buffer exchanged to 20 mM Hepes pH 7.5, 100 mM NaCl and 0.01% LMNG by SEC. Opsin N2C/M257Y/D282C mutant was prepared in the same way, but LMNG detergent was used post 1D4 resin binding.

**Expression and purification of mGα proteins**. N-terminal His-tagged mGα proteins were expressed in *E.coli* system and purified as previously described (4). Cells were lysed in buffer A (40 mM Hepes pH 7.5, 100 mM NaCl, 10% glycerol, 10 mM imidazole, 5 mM $MgCl_2$, 50 μM GDP) in presence of 100 μM DTT, protease inhibitors, DNAse and lysozyme. The supernatant

was loaded on a 10 ml Ni Sepharose FF column and washed with 10 CV of buffer B (20 mM Hepes pH 7.5, 500 mM NaCl, 40 mM imidazole, 10% glycerol, 1 mM $MgCl_2$, 50 µM GDP). Protein was eluted with 3 CV of buffer C (20 mM Hepes pH 7.5, 100 mM NaCl, 500 mM imidazole, 10% glycerol, 1 mM $MgCl_2$, 50 µM GDP). His-tag was cleaved off with TEV protease in presence of 1 mM DTT and the sample was simultaneously dialysed overnight against 2 L of buffer D (20 mM Hepes pH 7.5, 100 mM NaCl, 10% glycerol, 1 mM $MgCl_2$, 10 µM GDP). Sample was mixed for 2h after addition of 4 ml Ni-NTA resin and 20 mM imidazole and collected on a column containing additional 1 ml Ni-NTA resin. Flow-through was collected and the resin was washed with 2 CV of buffer D. Both fractions were pooled, concentrated to 1.5 ml and loaded onto a Superdex-200 26/600 gel filtration column equilibrated with buffer E (10 mM Hepes pH 7.5, 100 mM NaCl, 10% glycerol, 1 mM $MgCl_2$, 1 µM GDP, 0.1 mM TCEP). Peak fractions were pooled, concentrated, flash- frozen in liquid nitrogen and stored at -80°C. The method for expression and purification of the full-length mGα proteins and the truncated versions, mGo_Δ5 and mGi_Δ5, followed the same protocol.

**Expression and purification of Nb80**. Nanobody 80 was expressed and purified using modified previously described method (5). The Nb80 with a C-terminal His-tag was expressed in the periplasm of *E. coli* strain WK6. The cells were cultured in TB media supplemented with 0.1% (w/v) glucose and 2 mM $MgCl_2$. The expression was induced with 1 mM IPTG after OD600 of 0.70 was reached and the temperature was reduced to 28°C. The cells were harvested the next day and lyzed with ice-cold buffer (50 mM Tris-HCl pH 8.0, 12.5 mM EDTA, 0.125 M sucrose). After centrifugation, the protein was purified by nickel affinity chromatography followed by size-

exclusion chromatography on a Superdex75 60/300 column equilibrated with buffer 20 mM Hepes pH 7.45, 100 mM NaCl. The protein was concentrated to 100 mg/mL, flash frozen in liquid nitrogen and stored at -80°C.

**Preparation of heterotrimeric G protein subunits**. The full-length human Gαi subunit (G$\alpha i_1$) with an N-terminal deca-histidine tag was prepared by heterologous expression in *E. coli* strain BL21(DE3) and purified as described (6). The transducin heterotrimer (G$\alpha t\beta_1\gamma_1$) was isolated from the rod outer segment of bovine retina (W. L. Lawson Company) and Gβ1γ1 was separated from Gαt by Blue Sepharose 6 Fast Flow (GE Healthcare) (6).

**Preparation of β-arrestin-1**. The wild-type full-length human β-arrestin-1 with an N-terminal hexa-histidine tag was expressed and purified as described previously (7), but with minor modifications. Briefly, cells were lyzed by three cycles of sonication in solubilization buffer (50 mM Bis-Tris propane pH 7.0, 500 mM NaCl and 10% (v/v) glycerol). The lyzed cells was supplemented with 30 mM imidazole and purified using Ni-NTA FF crude column (GE Healthcare). The eluted protein was dialyzed overnight to a final salt concentration of 40 mM NaCl and subsequently applied onto a 15 ml Q sepharose column. The column was first washed with 20 mM Bis-Tris propane pH 7.0, 40 mM NaCl, 10% (v/v) glycerol and 14.3 mM 2-mercaptoethanol, followed by protein elution in 20 mM Bis-Tris propane pH 7.0, 200 mM NaCl, 10% (v/v) glycerol and 14.3 mM 2-mercaptoethanol. The protein was further purified by size-exclusion chromatography on a Superdex 200 Increase 10/300 GL column (GF Healthcare) equilibrated with 20 mM Bis-Tris propane pH 7.0, 300 mM NaCl, 10% (v/v) glycerol and 14.3

mM 2-mercaptoethanol. Peak fractions containing the protein was concentrated using a 30 kDa MWCO concentrator (Millipore) and stored at -80$^{o}$C until use.

**Preparation of purified proteins for mass spectrometry measurements**. In order to unify the reaction conditions, all proteins used for further measurements were buffer exchanged to Hepes buffer (20 mM, 0.01% LMNG, 40 mM NaCl, pH = 7.5), aliquoted to 10-20 µL, and stored in -20 °C, to prevent repeated freezing and thawing. Before mixing/incubating with other proteins/ligands, 0.4 mM TCEP was added to all protein solutions to ensure that the protein was present in monomeric form. The protein concentrations were measured by a UV NanoDrop 2000 / 2000c spectrometer (Thermo Fisher Scientific, Wilmington, USA).

**High-mass MALDI mass spectrometry of protein complexes**. A sandwich method (matrix/sample/matrix) was used to deposit the protein samples on a 384-spot MALDI plate (AB Sciex). First, the MALDI plate was cleaned by flushing/wiping alternately with methanol and Milli-Q water and finally with methanol. Then, a 1:500:500 (v/v/v) TFA/water/acetonitrile (TWA) solution and a 2:1:3 (v/v/v) formic acid/water/isopropanol (FWI) solution were prepared, respectively. A saturated sinapinic acid solution was prepared by dissolving SA in TWA, following by centrifuging at 14,000 rpm for 5 min to separate undissolved SA from the supernatant. Then a half-saturated SA solution was prepared by evenly mixing with FWI in a 1:1 ratio (v/v), which was deposited on the MALDI plate (0.5 µL/spot) and dried in air. This formed a uniform matrix crystal film on the surface of the plate, and such a matrix coated MALDI plate could be stored in clean air for several weeks if desired. Then, the protein samples were deposited on the plate (three

spots/sample), followed by drying in air. Finally, a saturated TWA solution of SA was deposited on top of the sample (0.5 µL/spot) and dried in air for high-mass MALDI-MS analysis. All mass spectrometric measurements were performed in linear positive ion mode on a MALDI-TOF/TOF mass spectrometer (model 4800 plus, AB Sciex, Darmstadt, Germany) equipped with a high-mass detector (HM2, CovalX AG, Zurich, Switzerland). The HV1 and HV2 voltages of the HM2 detector were set to −3.5 kV and −20.0 kV, respectively. MALDI was initiated by a Nd:YAG laser pulse (355 nm), and 500 shots per spectrum were accumulated. The Origin software was used to process the data, including plotting and normalisation of the mass spectra, calculation of the peak area, plotting of all the curves, fitting of the data and calculation of dissociation constants ($K_d$s).

**Determination of the number of mono-, intra- and inter-molecular links.** During the crosslinking, the three types of links (mono-, intra- and inter-molecular) are formed simultaneously according to the relative equilibrium of the species in the reaction. Once formed, these links act as locks on the protein. The unwanted links on the single protein components (receptor alone and partner protein alone) help to maintain the equilibrium between the formed complexes and single protein components by preventing further complex formation. As a result, there should be no further conformational changes due to complex formation after crosslinking.

The types of crosslinks on the protein after the crosslinking reaction was calculated using the following formulae, where ***a*** is monolink and ***b*** is intramolecular crosslink in the single protein components (GPCR or partner protein monomer), and ***a'*** is monolink, ***b'*** is intramolecular crosslink, and ***x*** is intermolecular crosslink in a GPCR•partner complex (based on the assumption

that the number of reacted lysine residues is same in the protein monomers and the protein complex):

$\boldsymbol{a}+2\boldsymbol{b}=\boldsymbol{a'}+2(\boldsymbol{b'}+\boldsymbol{x})$ [1]

The difference in the molecular weight (ΔMW=Δm/z) between m/z of the crosslinked complex and the total m/z of crosslinked GPCR and crosslinked partner protein was calculated:

i) if the NHS groups in the crosslinks are not hydrolyzed

$\Delta m/z=\boldsymbol{a}*MW_{(\text{crosslinker lost one -NHS})}+\boldsymbol{b}*MW_{(\text{crosslinker lost two -NHS})}-\boldsymbol{a'}*MW_{(\text{crosslinker lost one -NHS})}-(\boldsymbol{b'}+\boldsymbol{x})*MW_{(\text{crosslinker lost two -NHS})}$ [2]

ii) and if all the NHS groups are hydrolyzed

$\Delta m/z=\boldsymbol{a}*MW_{(\text{crosslinker lost two -NHS})}+\boldsymbol{b}*MW_{(\text{crosslinker lost two -NHS})}-\boldsymbol{a'}*MW_{(\text{crosslinker lost two -NHS})}-(\boldsymbol{b'}+\boldsymbol{x})*MW_{(\text{crosslinker lost two -NHS})}$ [2]'

Based on [1], [2], and [2]',

$\boldsymbol{x}=(\Delta m/z)/MW_{(\text{crosslinker or crosslinker lost two -NHS})}-(\boldsymbol{b'}-\boldsymbol{b})$ [3]

As the formation of inter- and intra-molecular crosslink are restricted by the distance of two lysine residues, we therefore assume the number of intra-molecular crosslinks in the GPCR•partner complex and the monomers are almost unchanged, so $\boldsymbol{b}-\boldsymbol{b'}=0$, and $\boldsymbol{x}=(\Delta m/z)/708.7$ (or 478.3) [4]

**Screening of ligand-mediated GPCR•partner complexes**. 10 μM ligand-mediated GPCR (apo-Rho, atr-Rho, apo-β1AR, iso-β1AR, S32212-β1AR, pro-β1AR, nad-β1AR, car-β1AR, apo-AT1R, angII-AT1R, or azi-AT1R; all ligands were added in molar excess, 2 mM) were mixed with equimolar amounts of partner protein (mGs, mGo, mGi, mGq, Nb80, mGo_Δ5, mGi_Δ5, or Gαi, Gαi•Gβ•Gγ and β-arrestin-1) and incubated for 1 hour at room temperature. The protein mixture

was then divided into two parts, one part was mixed with 5 mM BS(PEG)$_9$ at a ratio of 10: 1, v/ v, and crosslinked at room temperature for 1 hour, and the other part was mixed with Hepes buffer as a control. Finally, the GPCR mixtures were diluted to 2.5 μM and deposited on a MALDI plate via the sandwich method (three points/sample), followed by mass spectrometry analysis.

**Establishment of concentration standard curve**. Protein samples (Rho, β1AR, AT1R, Gαi, or mGo) were pre-treated with BS(PEG)$_9$ crosslinker for 1 hour at room temperature with the goal to block all lysine residues, and then diluted to gradient concentrations (*SI Appendix*, Fig. S5) using Hepes buffer. After being precooled on ice, the gradient concentrations of protein solutions were evenly and quickly mixed with precooled β-Galactosidase dissolved in Hepes buffer at a volume ratio of 1: 1, v/ v, respectively, and immediately deposited on the MALDI plate (three spots/sample) for further mass spectrometry analysis. Note that for obtaining the standard curves, different concentrations of β-Gal were chosen for different target proteins, i.e., 2.0, 0.6, 1.0, and 0.6 μM for rho, β1AR, AT1R, and mGo, respectively. Finally, the mass spectra were normalized by the intensity of β-Gal, and a standard curve was obtained by plotting the relative intensity (= analyte peak area/β-Gal peak area) of the analyte signals against the concentration.

**Calculation of dissociation constants ($K_d$s)**. 5 μM ligand-mediated GPCRs (apo-Rho, atr-Rho, apo-β1AR, iso-β1AR, apo-AT1R, angII-AT1R, or azi-AT1R) solutions were mixed with partner proteins (mGs, mGo, mGi, mGq, or Nb80, mGo_Δ5, or mGi_Δ5) at various concentration points respectively, followed by equilibration at room temperature for 1 hour. After BS(PEG)$_9$ crosslinking and the addition of β-Gal (the concentration of β-Gal for various GPCR is consistent

with the concentration in the standard curve), the protein mixtures were quickly deposited on the MALDI plate for further mass spectrometry analysis. Afterwards, the mass spectra were normalized by the intensity of the reference protein (β-Gal), and the concentrations of free GPCRs were calculated through the concentration standard curve. Eventually, the $K_d$s of the various GPCR•partner complexes were calculated using the following formulae:

GPCR + partner ⇋ GPCR•partner

$$[\text{GPCR•partner}] = [\text{GPCR}]_0 - [\text{GPCR}] \quad [5]$$

$$\frac{[\text{GPCR•partner}]}{[\text{GPCR}]_0} = 0.5\left[\left(K_d + [\text{GPCR}]_0 + [\text{partner}]_0\right) - \sqrt{\left(K_d + [\text{GPCR}]_0 + [\text{partner}]_0\right)^2 - 4[\text{partner}]_0[\text{GPCR}]_0}\right] \Big/ [\text{GPCR}]_0 \quad [6]$$

Where [GPCR] is the concentration of free GPCR at equilibration, $[\text{GPCR}]_0$ is the initial concentration of the GPCR, [GPCR•partner] is the concentration of GPCR•partner complex at the equilibration, and $[\text{partner}]_0$ is the initial concentration of partner protein. Equation [2] is valid if $K_d$ is less than ten times the total receptor concentration.

**Measurement of $K_d$ values with microscale thermophoresis (MST)**. 100 µl of 6 µM atr-Rho were mixed with 100 µl of 2nd generation RED-Maleimide dye at 18 µM and was incubated for 30 minutes in the dark at room temperature. The redundant dye was subsequently removed by gravitational flow chromatography. The labeling was performed in a buffer containing 20 mM Hepes, 160 mM NaCl and 0.01% LMNG. mGo was serially diluted from 100 µM to 3.05 nM with final volumes of 10 µl. 10 µl of a 10 nM labelled atr-Rho solution was added randomly to each dilution, resulting in final concentrations of 50 µM to 1.53 nM for the mGo and 5 nM of labelled atr-Rho. The experiments were performed in premium-coated capillaries (NanoTemper

Technologies, Munich, GER) on a Monolith NT.Pico (NanoTemper Technologies, Munich). Excitation power was set at 5% MST-power to 40% (equivalent to a temperature shift of 4 K) with a laser-on time of 20 sec and a laser-off time of 3 sec. Temperature was set to 25 °C. Each measurement was performed in triplicate. Ligand-induced fluorescence change was observed in all measurements. The data was analyzed for initial fluorescence with MO.Affinity (NanoTemper Technologies, Munich).

**Partner-protein competition and calculation of $K_i$.** Equimolar amounts of apo-β1AR, mGα protein (mGs, mGo, or mGq) and Nb80 were mixed, and incubated for 1 hour at room temperature. After $BS(PEG)_9$ crosslinking, the three-component protein mixtures were detected using high-mass MALDI-MS. Furthermore, a Nb80 titration experiment was done. Gradient concentrations of Nb80 solutions (final concentrations: 0.0, 0.83, 1.67, 3.33, 6.67, 10.0, 13.3 μM) were mixed with the mixture of iso-β1AR (final concentration 2.5 μM) and mGo (final concentration 3.0 μM), and allowed to react for 1 hour at room temperature. After crosslinking with $BS(PEG)_9$, the samples were mixed with β-Gal and analysed using high-mass MALDI-MS. The $EC_{50}$ and $IC_{50}$ values were calculated using Graphpad software 8.3.0 (*SI Appendix*, Fig. S14), and, the $K_i$ (Nb80) was calculated using the following formulae:

$EC_{50} = 1.58 \pm 0.22\ \mu M$, $IC_{50} = 4.6 \pm 0.8\ \mu M$ [7]

$K_i\ (Nb80) = IC_{50} / ([iso]_{fix} / EC_{50}+1) = 1.57\pm0.24\ \mu M$ [8]

$pK_i\ (Nb80) = -\log(K_i) = 5.8\pm0.9$ [9]

where $[mGo]_{fix}$ is the concentration of isoprenaline of the Nb80 titration experiment.

**Influence of ligands on the competition between Nb80 and mGo**. A mixture of 2.0 μM β1AR, 2.0 μM mGo, and 2.0 μM Nb80 was divided into seven parts, and mixed with gradient concentrations of isoprenaline respectively, final concentrations are 0.0, 0.8, 4.0, 20.0, 100.0, 500.0, and 1000.0 μM. After 1-hour reaction at room temperature, the above mixtures were crosslinked by $BS(PEG)_9$ and deposited on the MALDI plate for further detection.

**Ligand competition and calculation of inhibition constant ($K_i$)**. Gradient concentrations of isoprenaline solutions were prepared using the Hepes buffer, in order to titrate the mixtures of 5 μM apo-β1AR and 6 μM mGs (or Nb80), at the presence of 0, 2, and 50 μM inhibitor (s32212, nadolol, or (-)-propranolol), respectively. After the $BS(PEG)_9$ crosslinking and the addition of β-Gal, the protein mixtures (keep the final concentration of β1AR to be 2.5 μM) were analysed by high-mass MALDI-MS (*SI Appendix,* Fig. S17). In order to obtain $K_i$, the curve of β1AR•mGs formation percentage (= [β1AR•mGs]/$[\beta 1AR]_0$ x 100%) as a function of isoprenaline's concentration was plotted to obtain the $EC_{50}$ of isoprenaline (*SI Appendix,* Fig. S18 *B* and *C*). Furthermore, gradient concentrations of s32212 solutions were mixed with the mixture of apo-β1AR (2.5μM) and mGs (3.0μM), at the presence of 20 μM isoprenaline. The curve of the formation rate of β1AR•mGs against the concentration of s32212 was plotted (*SI Appendix,* Fig. S19) to get the $IC_{50}$ of s32212. $EC_{50}$ and $IC_{50}$ values were calculated by Graphpad software 8.3.0, and the $K_i$ of s32212 could be calculated using the following formulae:

$$EC_{50}\text{ (iso)} = 1.46 \pm 0.17\ \mu M,\ IC_{50}\text{ (s32212)} = 53 \pm 6\ \mu M \quad [10]$$

$K_i$ (s32212) = $IC_{50}$ / ($[iso]_{fix}$ / $EC_{50}$+1) = 3.56 ± 0.26 µM [11]

$pK_i$ (s32212) = -log ($K_i$) = 5.4 ± 0.4 [12]

where $[iso]_{fix}$ is the concentration of isoprenaline of the s32212 titration experiment.

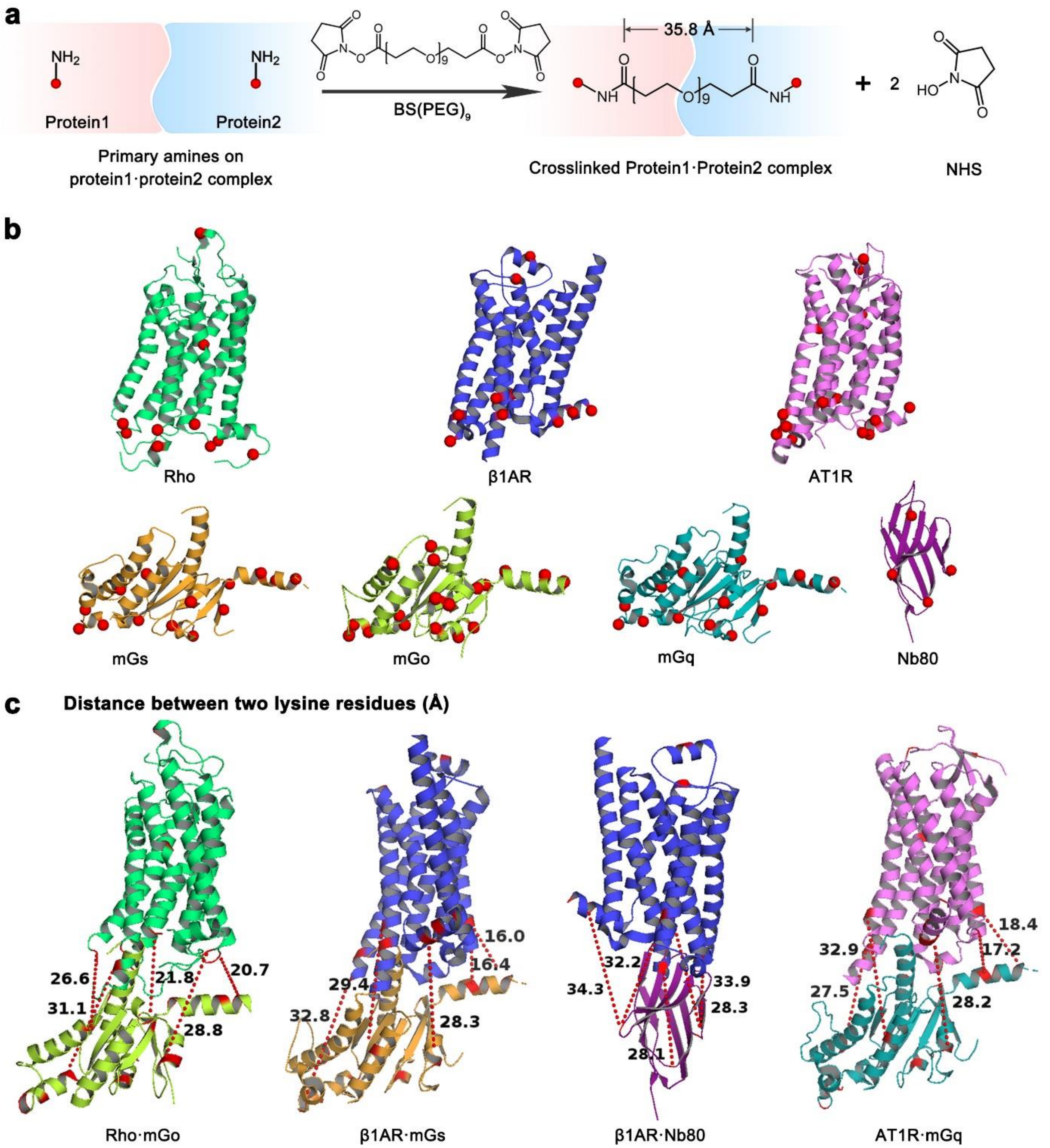


**Supplementary Fig. 1 | a,** Reaction of BS(PEG)$_9$ with lysine residues during crosslinking of proteins. **b,** Three-dimensional structural models of GPCRs and partner proteins and positions of lysine residues in each protein (red spheres). **c,** Distances in Å between lysine residues in GPCRs and partner proteins. Rho (PDB: 2I37), β1AR (PDB: 2Y03), AT1R (PDB: 6DO1), mGs (PDB: 3SN6), mGo (PDB: 6G79), mGq (homology model built with SWISS-MODEL (8) and using mGs as template), Nb80 (PDB: 3P0G).

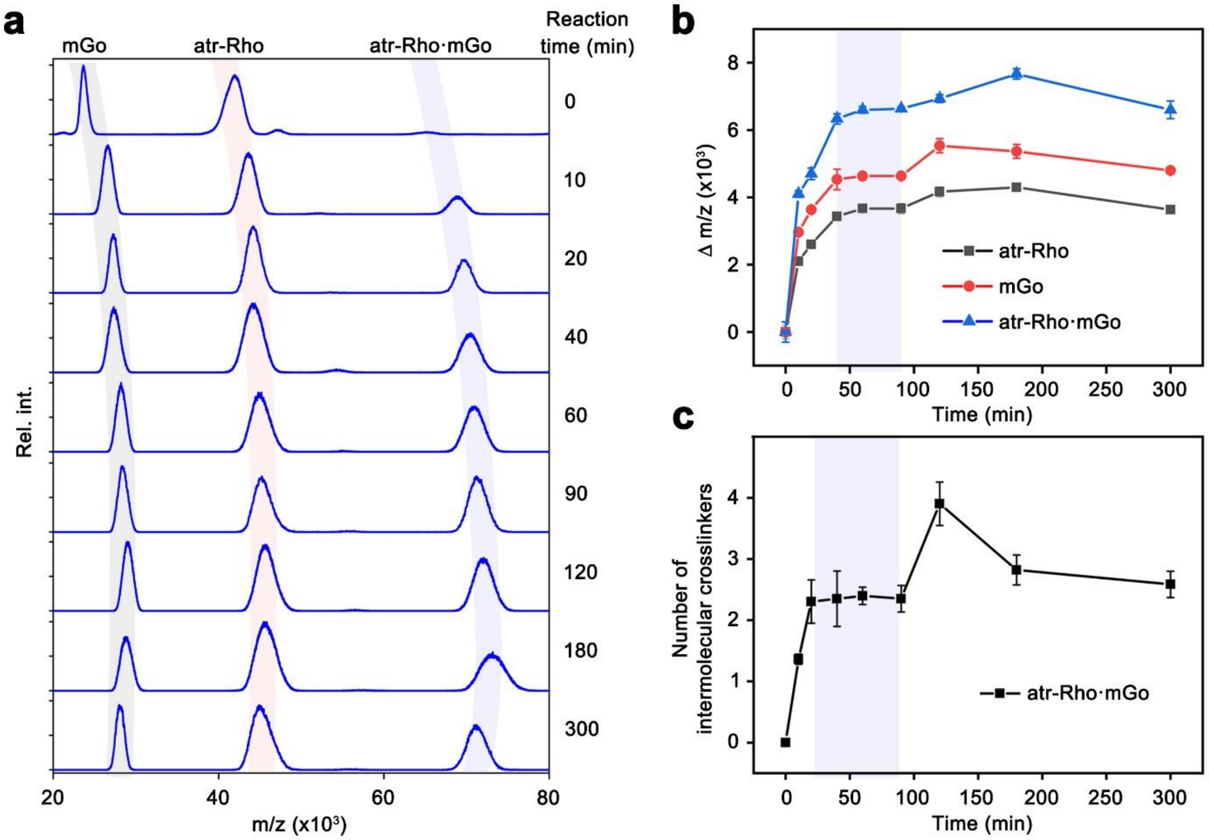


**Supplementary Fig. 2 |** Optimization of experimental conditions and crosslinking time using the all trans-retinal bound rhodopsin (atr-Rho) and mGo. **a,** Crosslinking rate of BS(PEG)$_9$, calculated by plotting the mass spectra of equilibrated atr-Rho and mGo mixtures stabilized by the crosslinker BS(PEG)$_9$ at various crosslinking times (0, 10, 20, 40, 60, 90, 120, 180, and 300 min). **b,** Peak shifts of atr-Rho, mGo, and atr-Rho•mGo. **c,** Amount of efficient crosslinkers of atr-Rho•mGo. The change of the m/z and the number of effective intermolecular crosslinkers reached a plateau from 40 to 90 minutes. Therefore, the crosslinking time for all experiments was kept between 60 and 90 min to ensure the effectivity and homogeneity of the crosslinking reaction. Each measurement was replicated three times; error bars represent standard deviations.

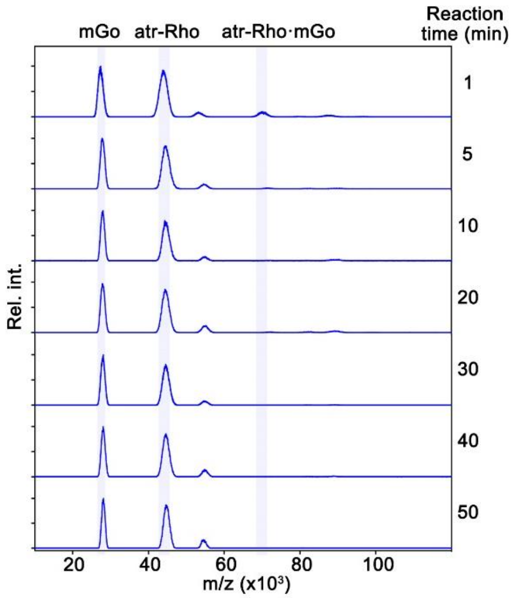


**Supplementary Fig. 3 |** Mass spectra of mixtures of atr-Rho and mGo after treating the proteins separately with crosslinker using different incubation times, from 1 to 50 min, prior to mixing. Almost no peak of atr-Rho•mGo (m/z: 71.8 ± 0.2 kDa, highlighted in blue) is present in the spectra, which shows that BS(PEG)$_9$ is able to block the binding site of the proteins in 1 minute, precluding protein-protein interaction.

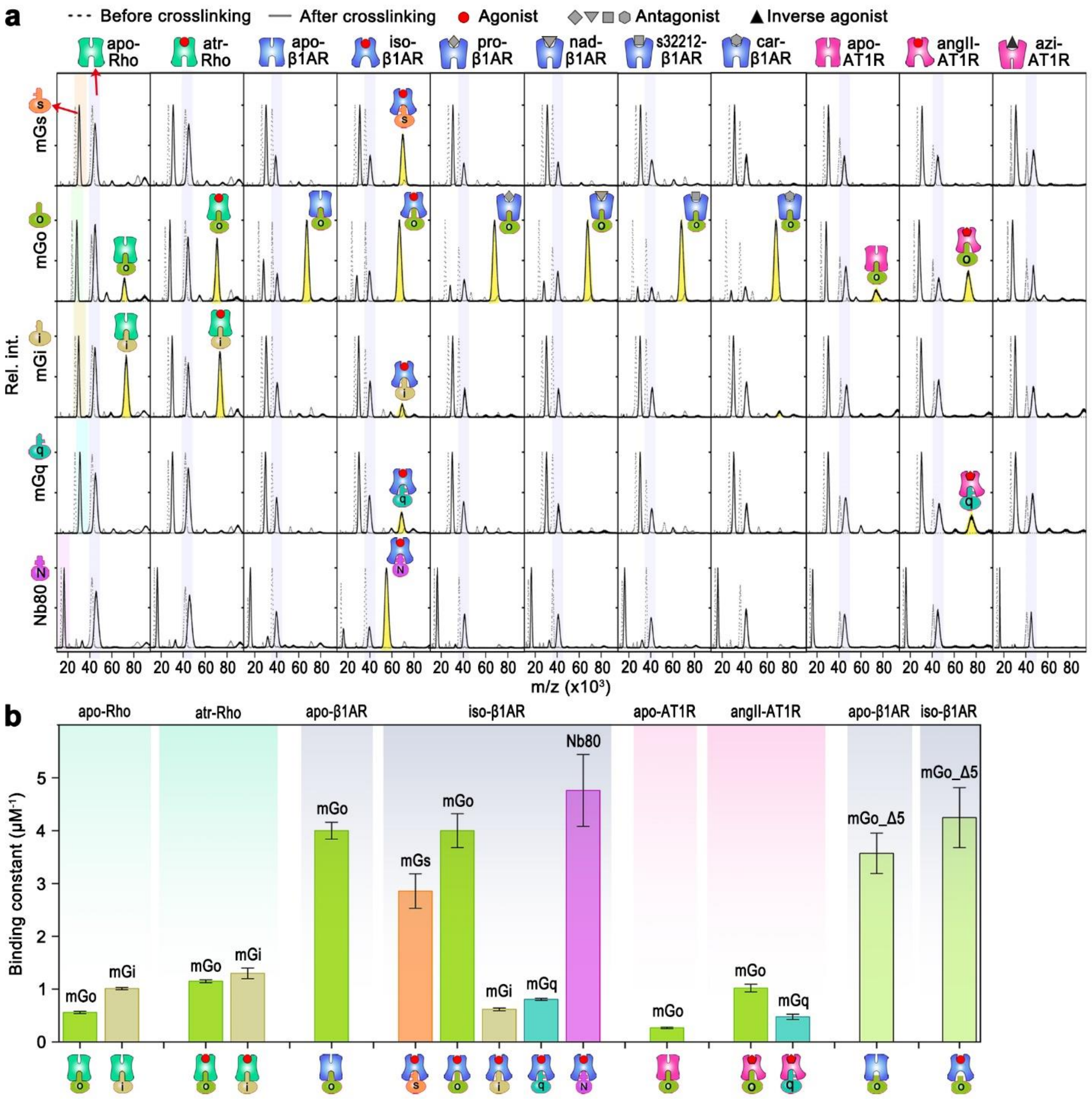


**Supplementary Fig. 4 | Selectivity in the complex formation of apo- and ligand-bound GPCRs with partner proteins assayed by high-mass MALDI-MS, and binding constants of the formed complexes**. **a,** Raw data showing complex formation of three GPCRs, rhodopsin (Rho), beta-1 adrenergic receptor (β1AR), and angiotensin II type 1 receptor (AT1R) in the presence or absence of agonists, antagonists or inverse agonists, with their partner proteins mGs, mGo, mGi, mGq and Nb80. The ligands used were the following: atr = all trans-retinal, iso = isoprenaline, pro = propranolol, nad = nadolol, s32212, car = carvedilol, angII = angiotensin II, and azi =azilsartan (see Supplementary Table 4). **b,** Comparison of the binding constants ($K_a$s) of the GPCR•partner protein complexes formed (shown in Fig. S4a). $K_a$ values were calculated using $K_d$ (Fig. 3c, Fig. 4b, and table S5) and the relationship $K_a=1/K_d$. Each measurement performed using at least 3 different incubations; error bars represent standard deviations.

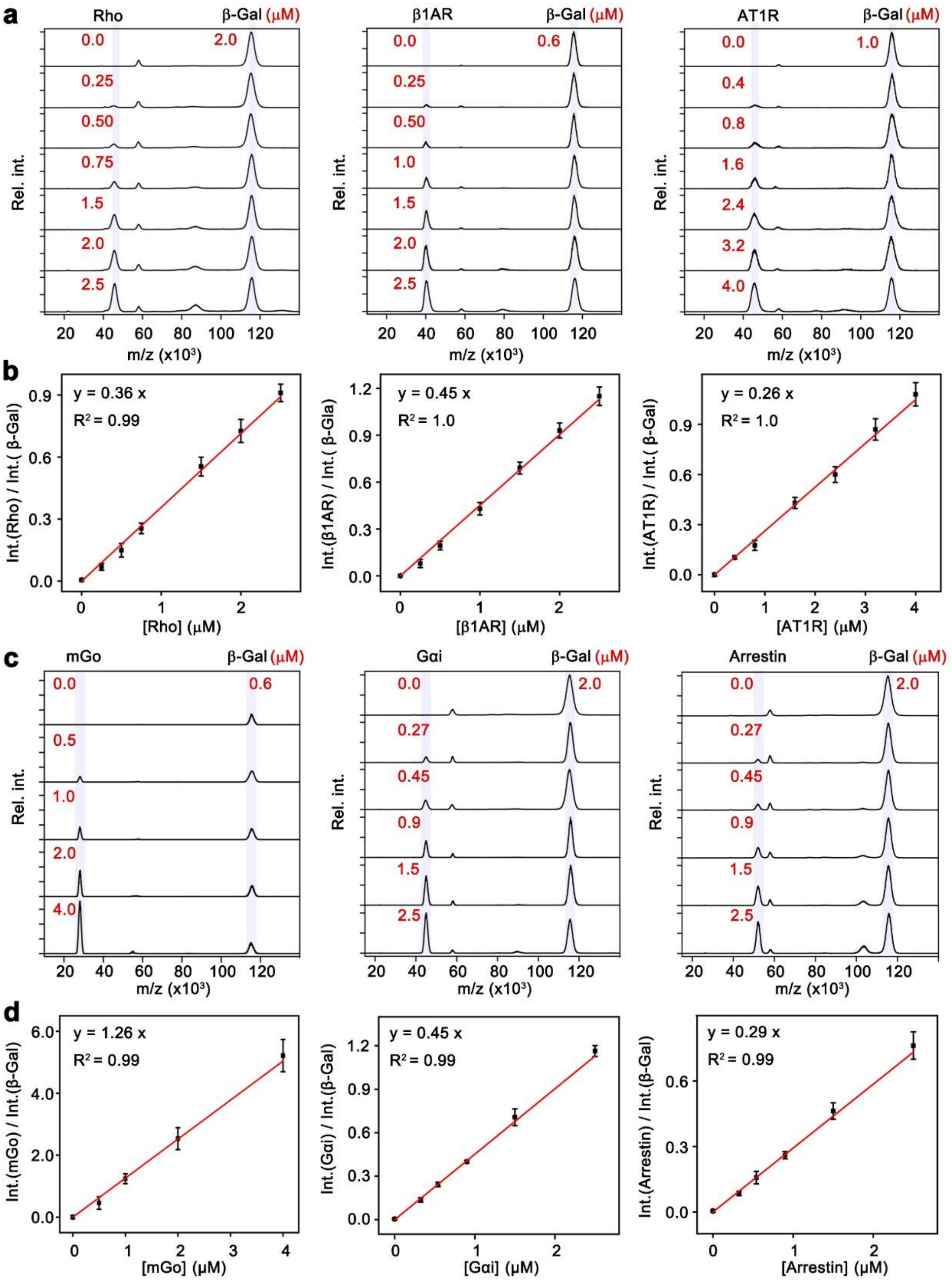


**Supplementary Fig. 5 | Protein standard curves for quantitative analysis of GPCR complexes. a-b,** Mass spectra of various concentrations of GPCRs mixed with fixed concentrations of β-Gal (2 μM, 0.6 μM, and 1 μM, respectively) and the corresponding calibration curves. **c-d,** Mass spectra of varying concentrations of mGo, Gαi and β-arrestin-1 after treatment with crosslinking agent, mixed with a fixed concentration of β-Gal (0.6 μM and 2.0 μM), and the corresponding calibration curves. Error bars represent standard deviation from three independent experiments.

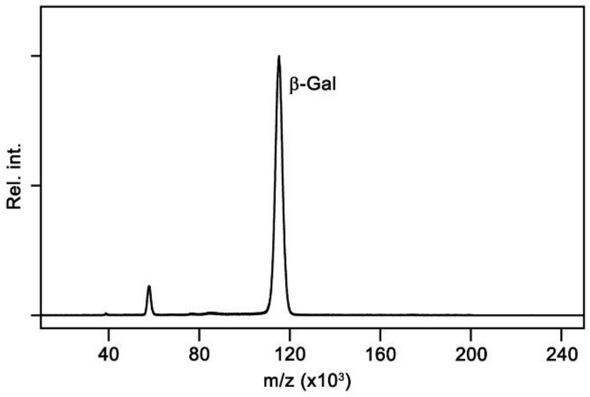


**Supplementary Fig. 6 |** Mass spectrum of $\beta$-galactosidase. The small signal at 58 kDa is the doubly charged peak of $\beta$-galactosidase.

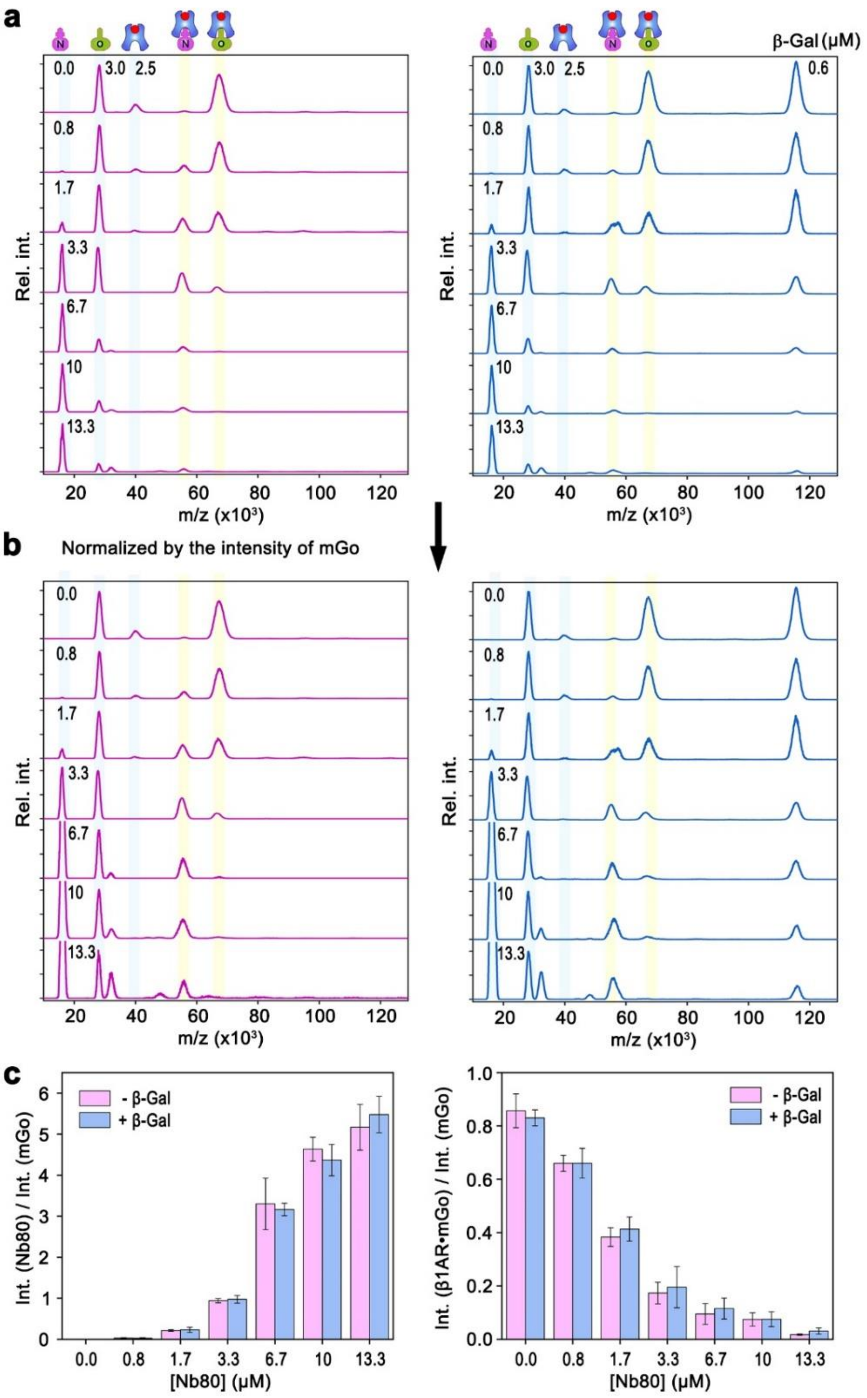


**Supplementary Fig. 7 | a,** Mass spectra showing titration of Nb80 to the mixture of mGo and β1AR, without the reference protein (left), and with the reference protein (right). **b,** Mass spectra normalised to the peak intensity of mGo, without the reference protein (left), and with the reference protein (right). **c,** Comparison of the changes in the peak intensity ratio of Nb80 to mGo (left) and the change in the peak intensity ratio of β1AR•mGo to mGo (right) in the mass spectra without reference protein (pink) and with reference protein (light blue).

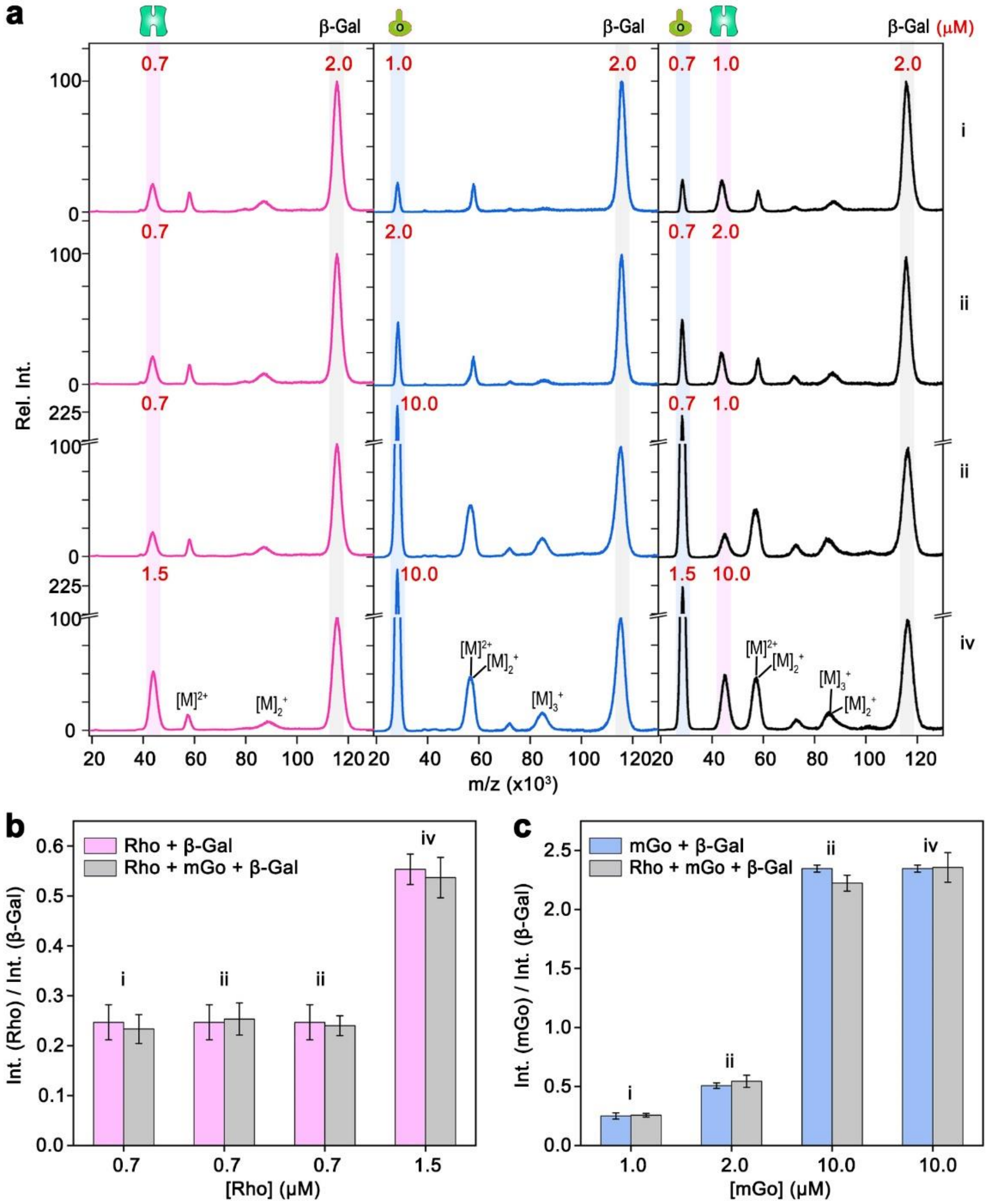


**Supplementary Fig. 8 | Comparison of the change of peak intensity ratio in the two-component system and the three-component system. a,** Mass spectra of different concentrations of Rho (0.7, 0.7, 0.7, and 1.5 μM) mixed with 2.0 μM β-Gal (first column), different concentrations of mGo (1.0, 2.0, 10, and 10 μM) mixed with 2.0 μM β-Gal (middle column), and different concentrations of the two components (third column, from top to bottom: 0.7/0.7/2.0, 2.0/0.7/2.0, 10/0.7/2.0, and 10/1.5/2.0 μM, mGo/Rho/β-Gal). **b,** Comparison of the intensity ratio of Rho to β-Gal in **a** (first column and third column). **c,** Comparison of the intensity ratio of mGo to β-Gal in **a** (middle column and third column). Error bars were calculated from three technical repeats.

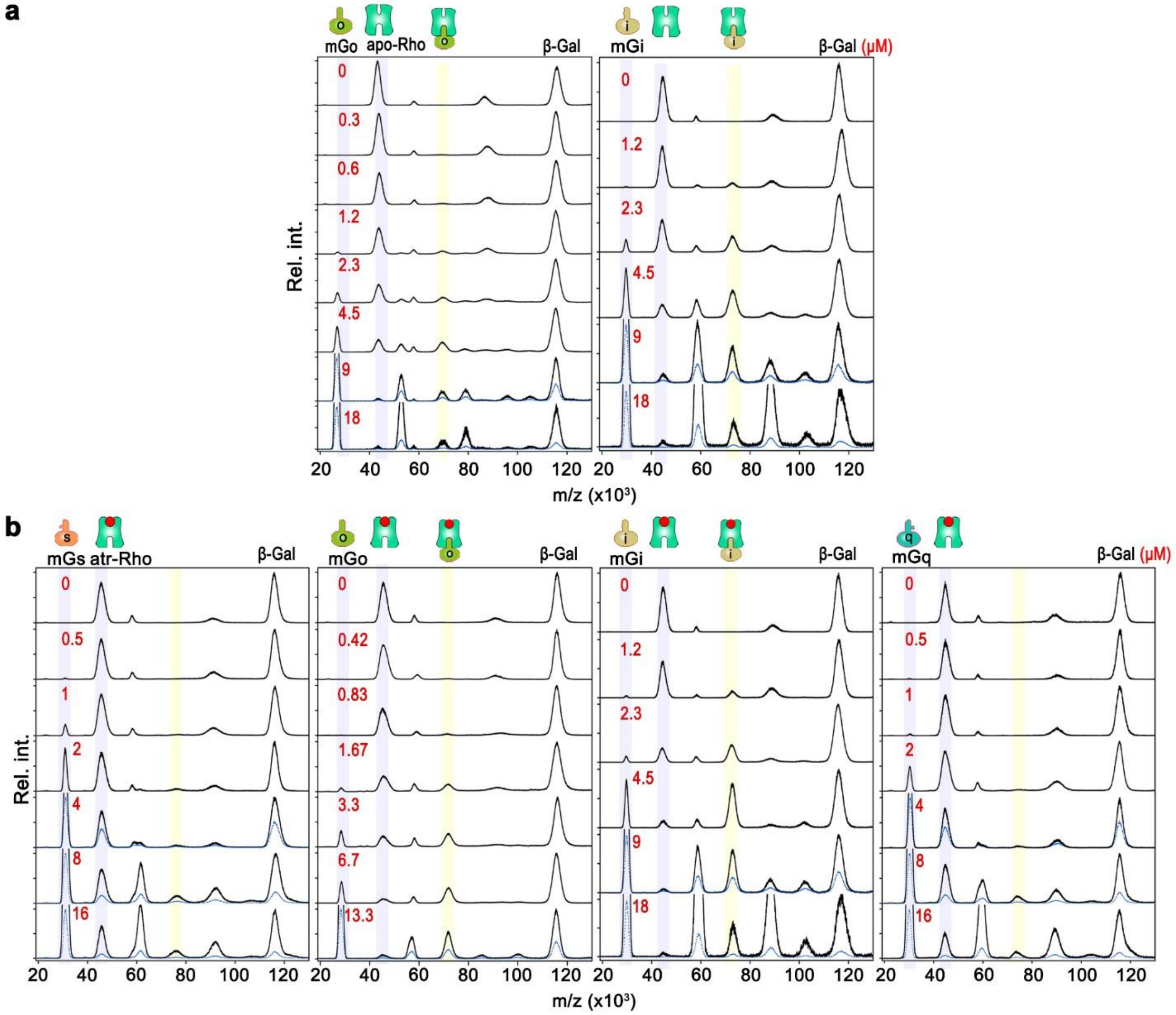


**Supplementary Fig. 9 | a,** Mass spectra of the formation of apo-Rho•partner complexes with the titration of mGo and mGi, and **b,** of atr-Rho•partner complexes with the titration of mGs, mGo, mGi, and mGq (from left to right). 2.0 μM β-Gal was used as the internal standard protein.

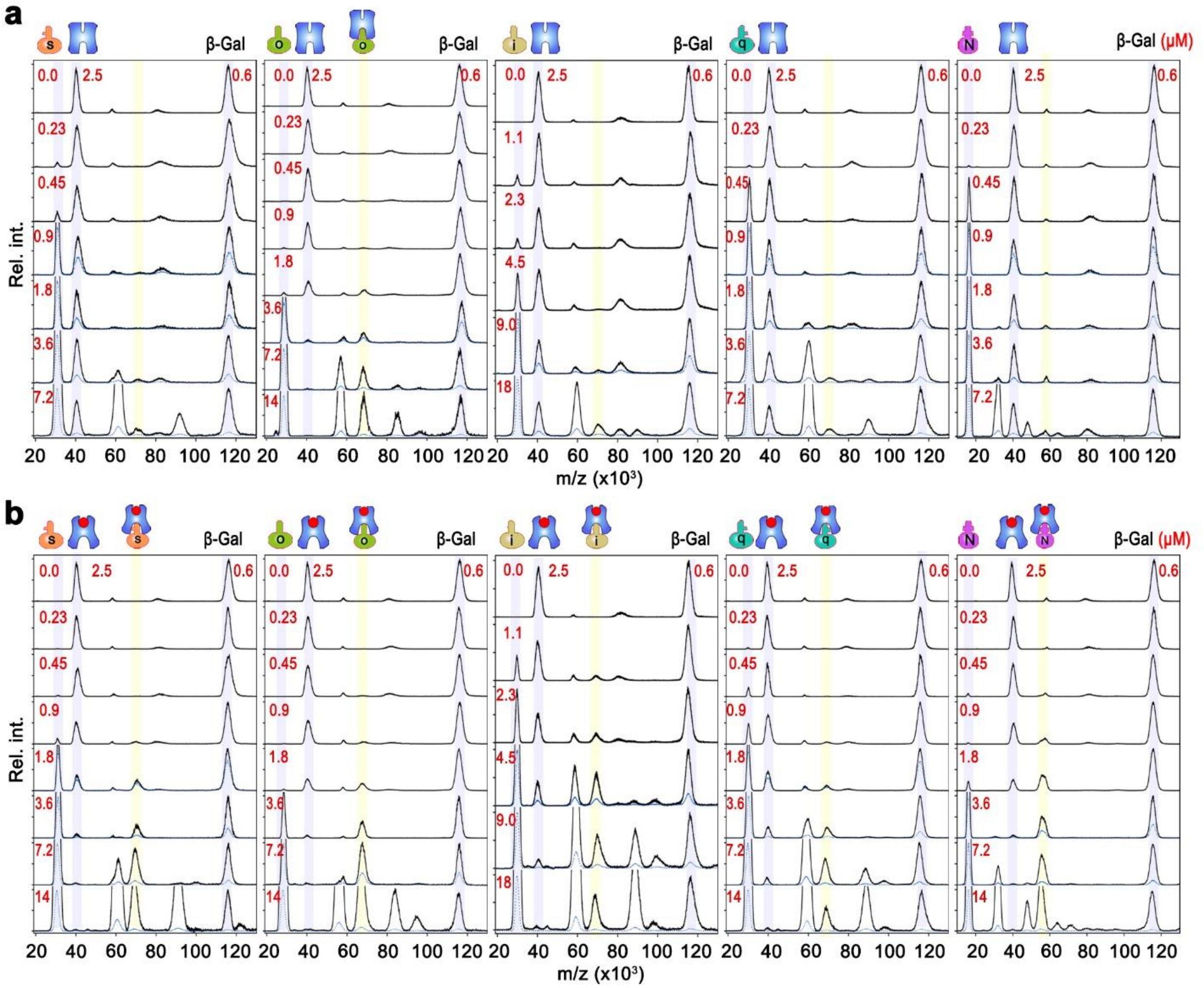

**Supplementary Fig. 10 | a,** Mass spectra of the formation of apo-β1AR•partner complexes and **b,** of iso-β1AR •partner complexes with the titration of mGs. mGo, mGi, mGq, and Nb80 (from left to right). 0.6 μM β-Gal was used as the internal standard protein.

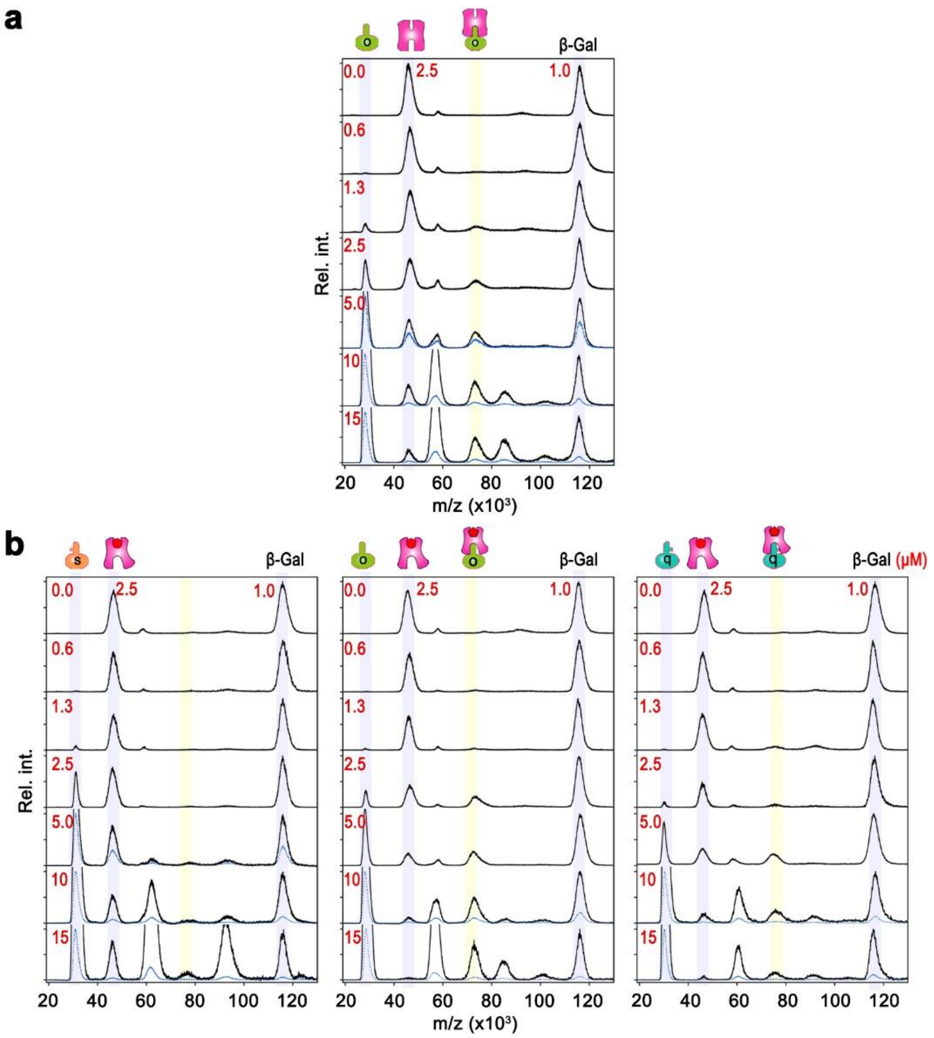


**Supplementary Fig. 11 | a,** Mass spectra of the formation of apo-AT1R•partner complexes with titration of mGo, and **b,** angII-AT1R•partner complexes with titration of mGs. mGo, and mGq (from left to right). 1.0 μM β-Gal was used as the internal standard protein.

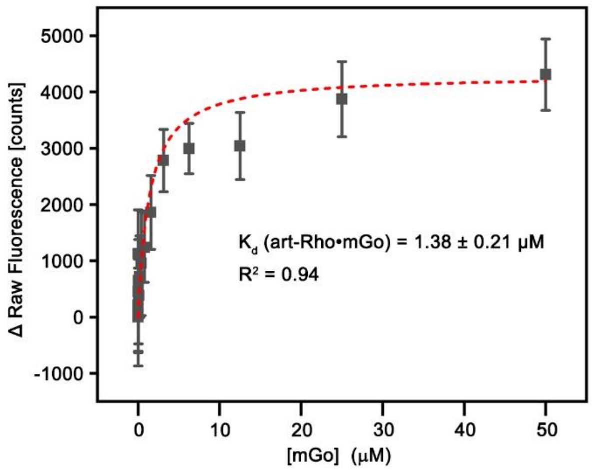


**Supplementary Fig. 12 |** Example of a dissociation constant ($K_d$) measurement via microscale thermophoresis (MST), with three technical repeats. The average $K_d$ was calculated, including error propagation, and for of Rho•mGo was $K_d$ = 1.38 ± 0.21 μM, which is calculated by the formula: Δ Raw fluorescence = (Δ Raw fluorescence)$_{max}$/($K_d$ + [mGo]).

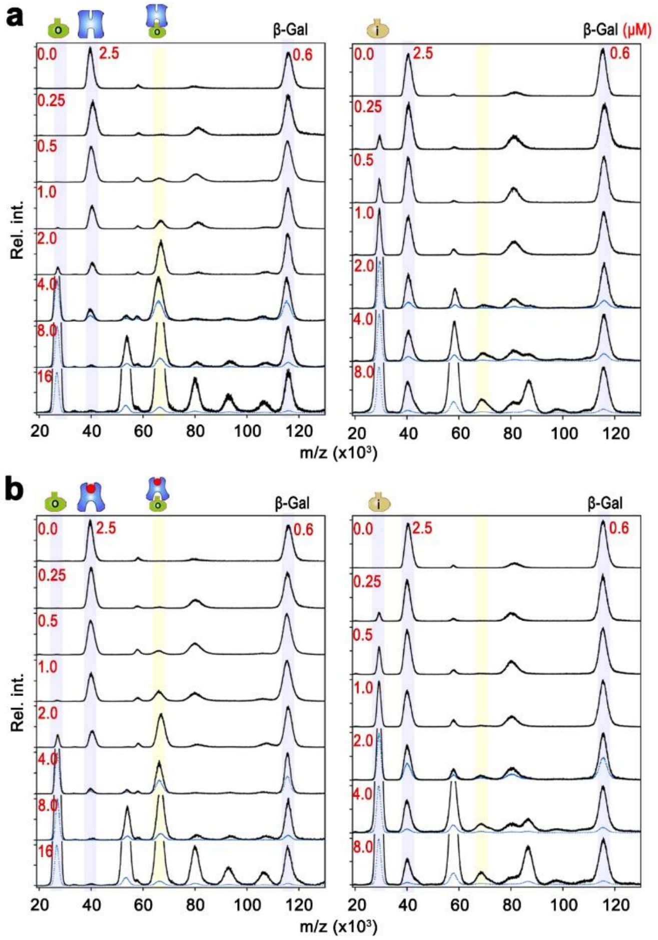


**Supplementary Fig. 13 |** Mass spectra of the formation of: **a,** apo-β1AR• mGo_Δ5 and apo-β1AR• mGi_Δ5 complexes with the titration of mGo_Δ5 and mGi_Δ5, from left to right; **b,** Same experiment in the presence of isoprenaline. 0.6 μM β-Gal was used as the internal standard protein.

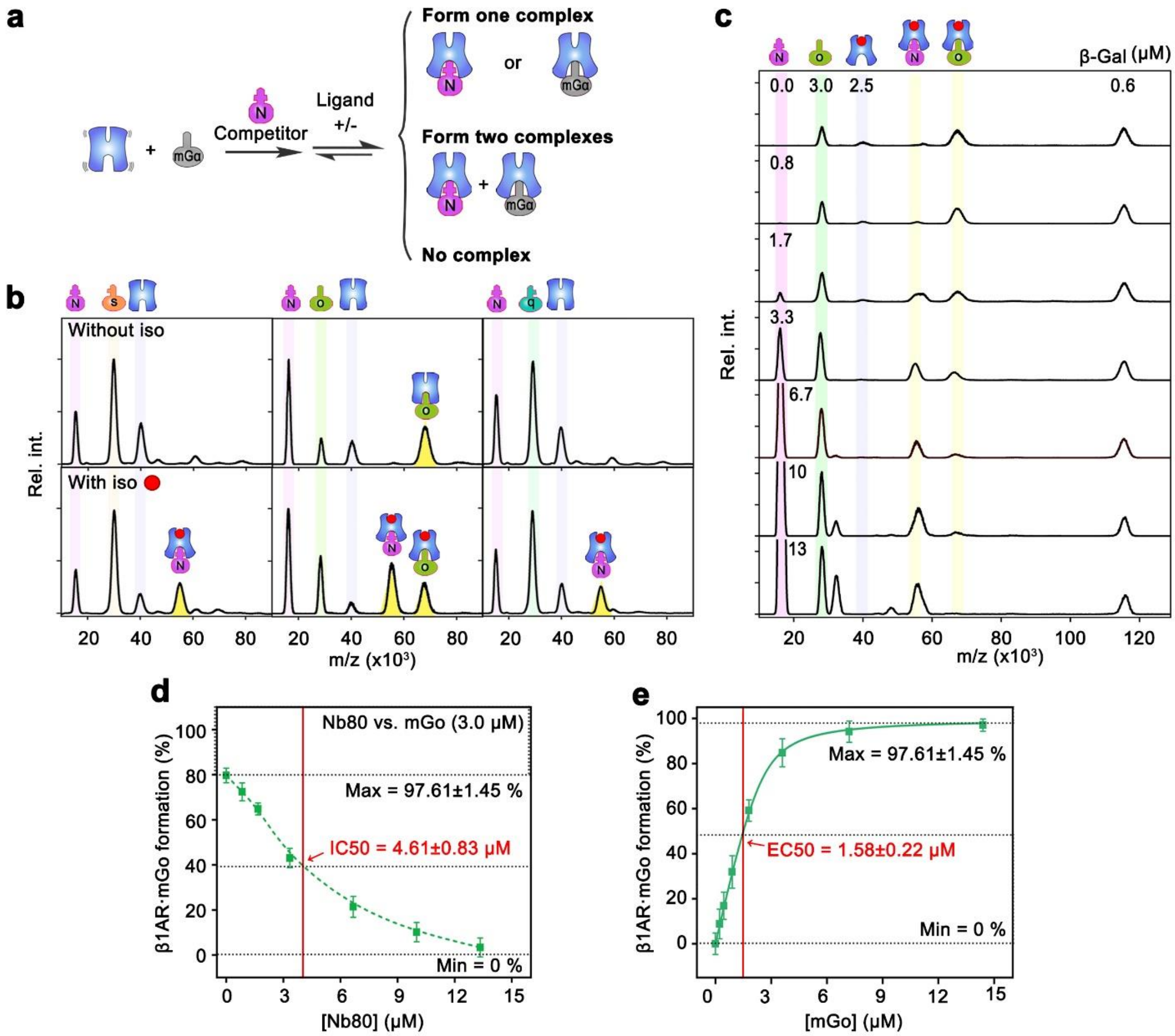


**Supplementary Fig. 14 | Competition between Nb80 and mGα proteins for GPCRs, and calculation of the Ki of Nb80. a,** Schematic of the competition between Nb80 and other mini-Gα proteins (mGs, mGo, and mGq) while binding to ligand-bound β1AR, and the different assembly possibilities. **b,** Mass spectra of the assembly of β1AR•partner complexes in the presence of equimolar concentrations of Nb80 and mGα proteins (from left to right, mGs, mGo, and mGq are shown), without isoprenaline (top row), and with isoprenaline (bottom row). **c,** Mass spectra showing the titration of Nb80 with 2.5 μM β1AR and 3.0 μM mGo, and conversion from β1AR•mGo to β1AR•Nb80. **d,** Formation of β1AR•mGo with the titration of NB80, and the reading of $IC_{50}$. **e,** Formation of β1AR•mGo with the titration of mGo and the reading of $EC_{50}$. The $K_i$ was calculated as explained in Online Methods: $K_i = IC_{50} / ([mGo]_{fix} / EC_{50}+1) = 1.57\pm0.24$ μM. Each measurement was replicated three times; error bars represent standard deviations.

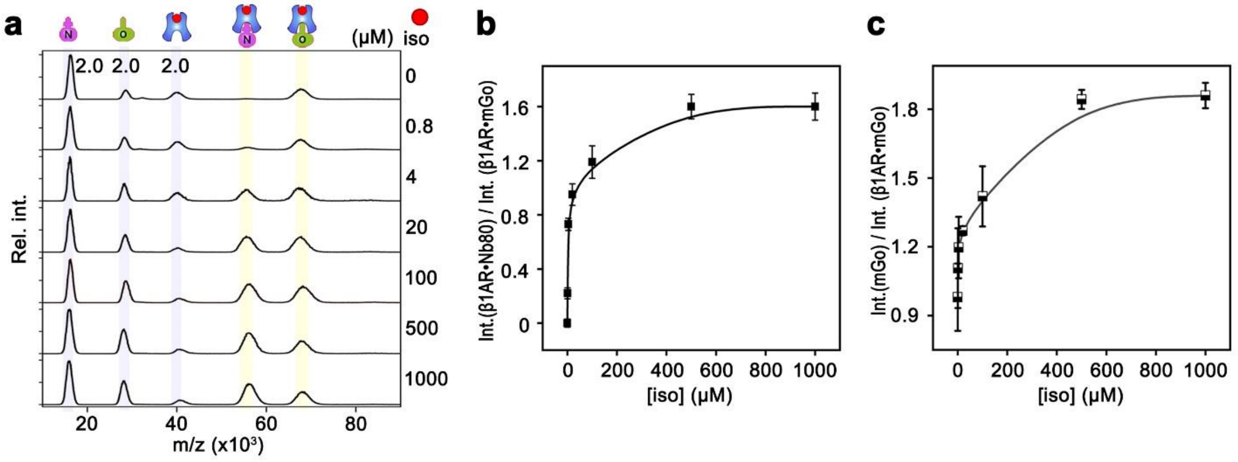


**Supplementary Fig. 15 | a,** Mass spectra of the concentration of the conversion between β1AR•mGo and β1AR•Nb80 modulated by isoprenaline. **b,** Intensity ratio of β1AR•Nb80 to β1AR•mGo as a function of added isoprenaline, based on the data shown in **a**. **c**, Intensity ratio of mGo to β1AR•mGo, as a function of added isoprenaline, based on the data shown in **a**. Each measurement was replicated three times; error bars represent standard deviations.

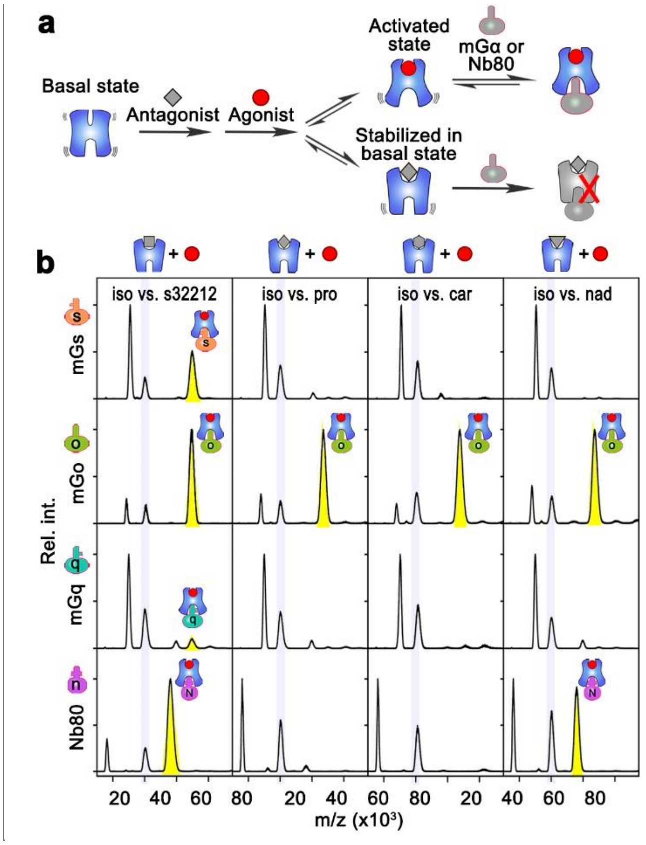


**Supplementary Fig. 16 | Binding of partner proteins to β1AR as a function of ligand competition. a,** Schematic of GPCR conformational ensembles induced by the competition between antagonist and agonist. **b,** Mass spectra of the selective assembly of ligand-bound GPCR (first column: β1AR/S32212/iso at 2.5/50/50 μM, respectively; second column: β1AR/pro/iso at 2.5/50/50 μM, respectively; third column: β1AR/car/iso at 2.5/50/50 μM, respectively; fourth column: β1AR/nad/iso at 2.5/50/50 μM respectively) with various partner proteins (from top to bottom: mGs, mGo, mGq, and Nb80).

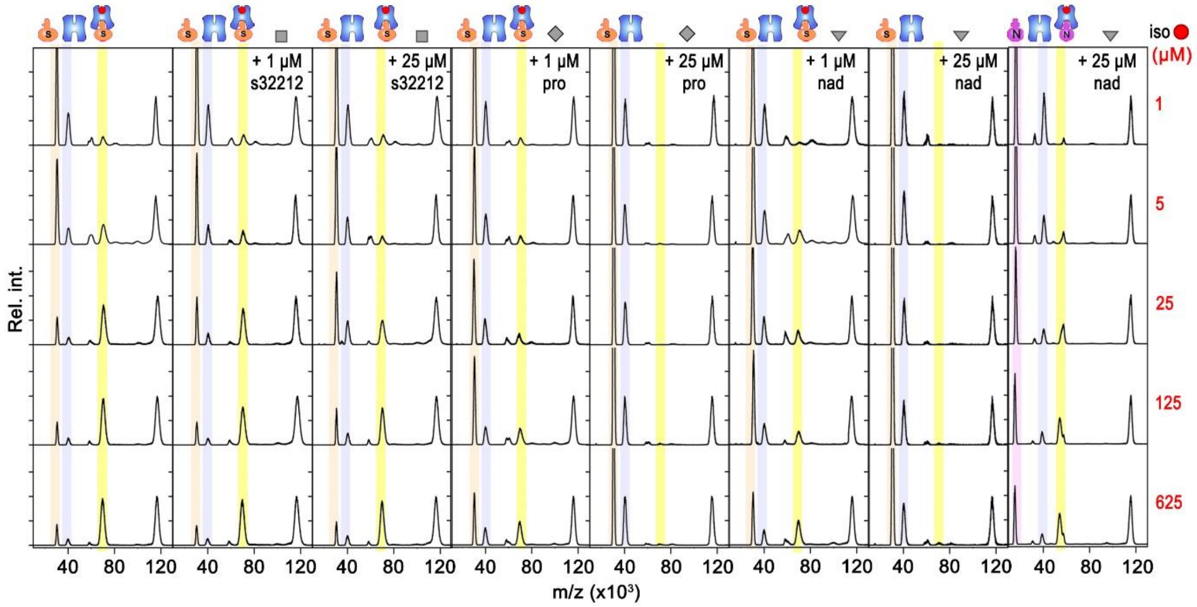


**Supplementary Fig. 17 | Competition between agonist (isoprenaline) and antagonists (S32212, propranolol, and nadolol)**. Formation of β1AR•mGs as a function of isoprenaline concentration and in the presence of 1 μM S32212 (second column), 25 μM S32212 (third column) 1 μM (-)-propranolol (fourth column), 25 μM (-)-propranolol (fifth column), 1 μM nadolol (sixth column), and 25 μM nadolol (seventh column). The last column shows formation of β1AR•Nb80 in the presence of 25 μM nadolol. 0.6 μM β-Gal was used as the internal standard protein.

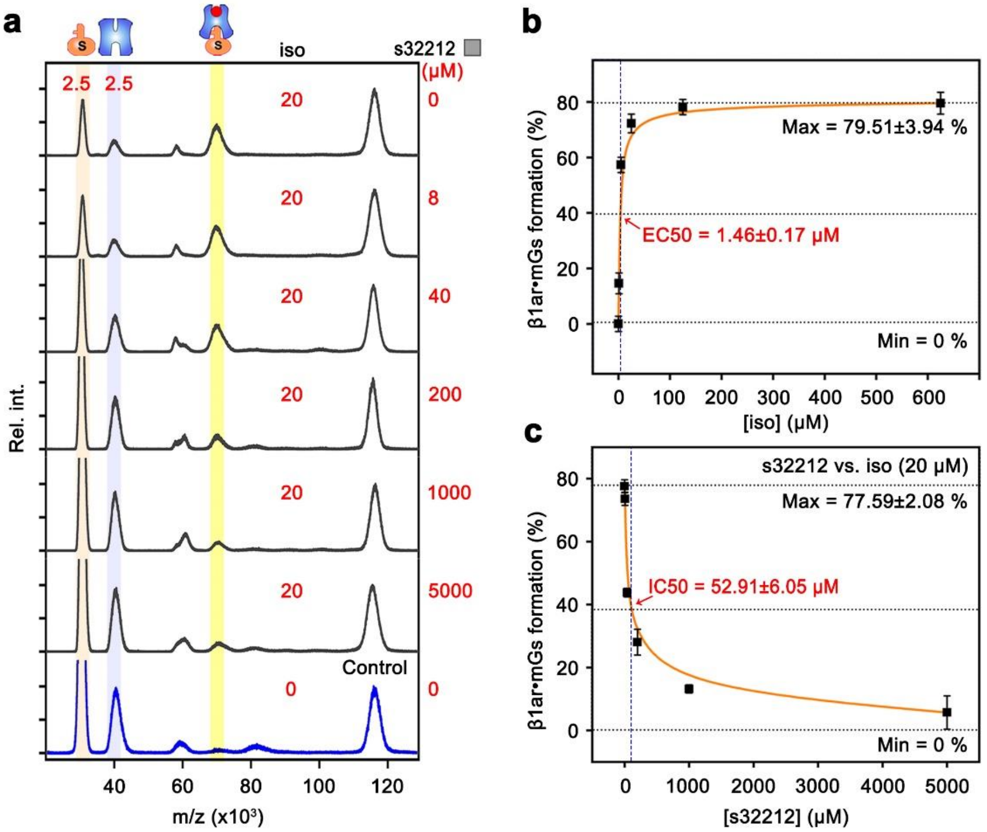


**Supplementary Fig. 18 | Calculation of the Ki of s32212**. **a,** Mass spectra of the titration of s32212 in competition with iso showing the decrease in the formation of β1AR•mGs. **b,** Assembly of β1AR•mGs vs. concentration of isoprenaline based on the data shown in Fig. S17. **c,** Assembly of β1AR•mGs vs. concentration of s32212. The $K_i$ is calculated as explained in Online Methods: $K_i = IC_{50} / ([iso]_{fix} / EC_{50}+1) = 3.56\pm0.26$ µM. Each measurement was replicated three times; error bars represent standard deviations.

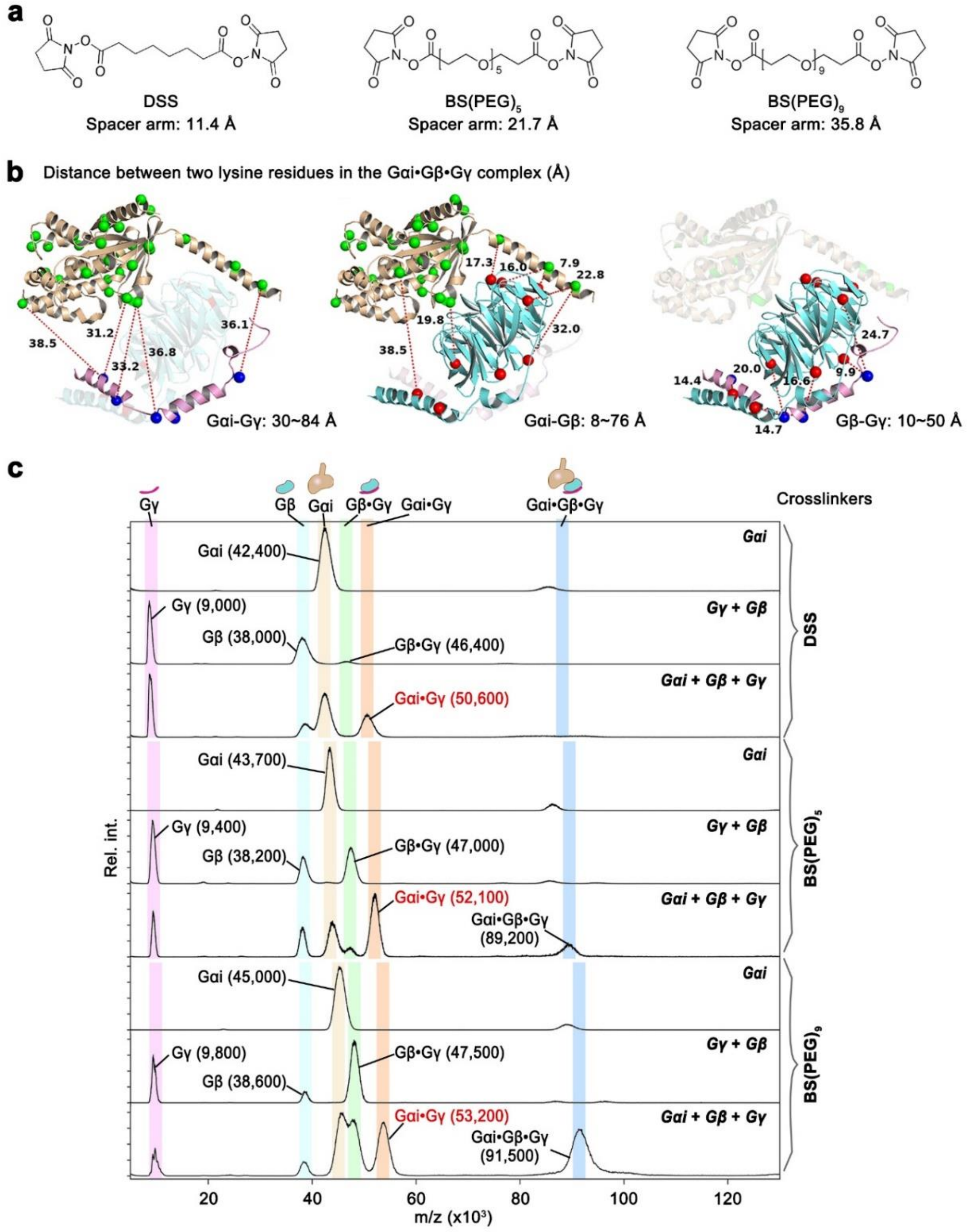


**Supplementary Fig. 19 | Monitoring the formation of Gαi•Gγ complex using chemical crosslinkers of increasing lengths and high-mass MALDI-MS. a,** Chemical structures of the crosslinkers and their spacer arm lengths. **b,** Structure of a heterotrimeric G protein (PDB code: 1GP2) and the measured distances in Å between lysine residues in the G protein subunits. Lysine residues are highlighted as green, blue, and red spheres. **c,** Mass spectra of complexes formed between G protein subunits following incubation and crosslinking with DSS, BS(PEG)$_5$ or BS(PEG)$_9$. Distances of lysines between Gαi and Gγ subunits in the heterotrimeric G protein were measured to lie between 30 and 84 Å. Using shorter crosslinkers we were still able to detected complexes of Gαi and Gγ, suggesting that the Gαi•Gγ complex exists independently of Gβ.

**Supplementary Table 1 |** Molecular weight/charge (m/z) increase of GPCRs and partner proteins before and after crosslinking, and the maximum number of crosslinker molecules bound to the proteins, assuming that there are no hydrolysis of the NHS group.

| Monomer protein | m/z (*$10^3$) | | Δ m/z (*$10^3$) | Number of crosslinked lysine residues* | Total number of lysine residues present |
|---|---|---|---|---|---|
| | **Before crosslinking** | **After crosslinking** | | | |
| **Crosslinker: BS(PEG)$_9$ (MW = 708.7 Da, MW$_{\text{(lost one -NHS group)}}$ = 593.5 Da, MW$_{\text{(lost two -NHS group)}}$ = 478.3 Da)** | | | | | |
| Rho | 41.5 ± 0.2 | 45.2 ± 0.2 | 3.7 | 6~15 | 11 |
| β1AR | 35.8 ± 0.1 | 39.9 ± 0.2 | 4.1 | 7~17 | 12 |
| AT1R | 40.7 ± 0.1 | 45.9 ± 0.3 | 5.2 | 9~22 | 19 |
| mGs | 26.3 ± 0.1 | 30.6 ± 0.2 | 4.3 | 7~18 | 14 |
| mGo | 23.6 ± 0.1 | 28.3 ± 0.2 | 4.7 | 8~20 | 18 |
| mGi | 26.2 ± 0.1 | 30.5 ± 0.2 | 4.4 | 7~18 | 15 |
| mGq | 26.3 ± 0.1 | 30.7 ± 0.2 | 4.4 | 7~18 | 15 |
| Nb80 | 14.2 ± 0.1 | 16.5 ± 0.1 | 2.3 | 4~9 | 4 |
| Gαi | 40.5 ± 0.1 | 45.0 ± 0.2 | 4.5 | 8~19 | 30 |
| Gγ | 8.3 ± 0.1 | 9.8 ± 0.2 | 1.5 | 3~6 | 10 |
| Gβ | 37.2 ± 0.1 | 38.6 ± 0.2 | 1.4 | 2~6 | 10 |
| **Crosslinker: BS(PEG)$_5$ (MW = 532.5 Da, MW$_{\text{(lost one -NHS group)}}$ = 417.3 Da, MW$_{\text{(lost two -NHS group)}}$ = 302.1 Da)** | | | | | |
| Gαi | 40.5 ± 0.1 | 43.7 ± 0.3 | 3.2 | 8~21 | 30 |
| Gγ | 8.3 ± 0.1 | 9.4 ± 0.2 | 1.1 | 3~7 | 10 |
| Gβ | 37.2 ± 0.1 | 38.2 ± 0.2 | 1.0 | 2~7 | 10 |
| **Crosslinker: DSS (MW = 368.4 Da, MW$_{\text{(lost one -NHS group)}}$ = 253.2 Da, MW$_{\text{(lost two -NHS group)}}$ = 138 Da)** | | | | | |
| Gαi | 40.5 ± 0.1 | 42.4 ± 0.2 | 1.9 | 8~28 | 30 |
| Gγ | 8.3 ± 0.1 | 9.0 ± 0.2 | 0.7 | 3~10 | 10 |
| Gβ | 37.2 ± 0.1 | 38.0 ± 0.2 | 0.8 | 3~12 | 10 |

*Only monolinker presence: number of crosslinked lysine residues = [m/z (after crosslinking) – m/z (before crosslinking)]/MW$_{\text{(crosslinker lost one -NHS)}}$
Only intra-molecualr crosslinker presence: number of crosslinked lysine residues = 2*[m/z (after crosslinking) – m/z (before crosslinking)]/MW$_{\text{(crosslinker lost two -NHS)}}$

**Supplementary Table 2 |** Number of intermolecular crosslinks present in each complex. BS(PEG)$_9$ was used as the crosslinker.

| GPCR Complex | m/z (GPCR + partner, crosslinked) (*$10^3$) | m/z (GPCR• partner) (*$10^3$) | Δ m/z (*$10^3$) | Number of intermolecular crosslinks * |
|---|---|---|---|---|
| Rho•mGo | 73.5 ± 0.4 | 71.8 ± 0.2 | 1.6 | 2~3 |
| Rho•mGi | 75.7 ± 0.4 | 73.8 ± 0.2 | 1.5 | 2~3 |
| β1AR•mGs | 71.2 ± 0.2 | 69.0 ± 0.3 | 1.5 | 2~3 |
| β1AR•mGo | 68.7 ± 0.4 | 67.0 ± 0.4 | 1.7 | 2~4 |
| β1AR•mGi | 71.1 ± 0.4 | 69.5 ± 0.2 | 1.6 | 2~3 |
| β1AR•mGq | 70.9 ± 0.4 | 69.4 ± 0.2 | 1.5 | 2~3 |
| β1AR•Nb80 | 56.5 ± 0.3 | 55.6 ± 0.3 | 1.4 | 2~3 |
| β1AR•Gαi | 85.2 ± 0.4 | 82.8 ± 0.3 | 2.4 | 3~5 |
| AT1R•mGo | 74.1 ± 0.5 | 72.6 ± 0.2 | 1.5 | 2~3 |
| AT1R•mGq | 76.3 ± 0.5 | 74.9 ± 0.2 | 1.4 | 2~3 |
| Gαi•Gγ | 54.6 ± 0.4 | 53.2 ± 0.4 | 1.4 | 2~3 |
| Gγ•Gβ | 48.4 ± 0.4 | 47.5 ± 0.3 | 0.9 | 1~2 |

*The calculation is based on the molecular weight of BS(PEG)$_9$ = 708.7 Da, and a net mass addition of 478.3 Da for each fully reacted or hydrolyzed BS(PEG)$_9$ molecule. Assuming that the number of lysine residues that have reacted are same in the protein monomers and the protein complex, either none (MW =708.7 Da) or all (MW = 478.3 Da) of the NHS groups is hydrolyzed after a 1-hour reaction time, the number of intermolecular crosslinks is calculated as follows:

[m/z (crosslinked GPCR) + m/z (crosslinked partner protein) – m/z (crosslinked complex)] / 708.7 (or 478.3)

**Supplementary Table 3 |** Chemical structures and properties of GPCR ligands used in this study, and catalog of cartoons used throughout this work.The pKi values were obtained from IUPHAR/BPS Guide to Pharmacology (9).

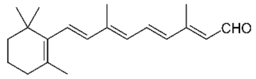

all-*trans* retinal
MW = 284.4 Da
full agonist for Rho

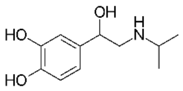

isoprenaline
MW = 211.3 Da
full agonist
for β1AR
($pK_i$=6.6-7.0)

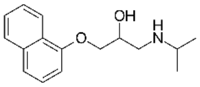

propranolol
MW = 259.3 Da
antagonist
for β1AR
($pK_i$ =8.20-8.7)

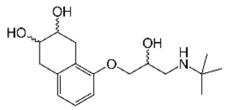

nadolol
MW = 309.4 Da
antagonist
for β1AR
($pK_i$ =7.23-8.02)

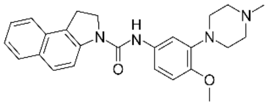

s32212
MW = 416.5 Da
(antagonist for a2AR
and β1AR)
($pK_i$ = 5.35)

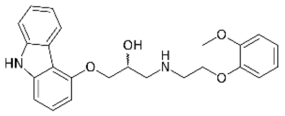

carvedilol
MW = 406.5 Da
antagonist/biased
agonist for β1AR
($pK_i$ = 9.5)

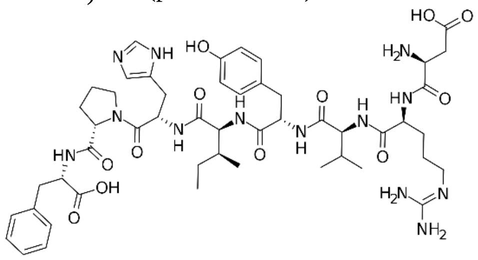

angiotensin II
MW = 1046.2 Da
full agonist for AT1R

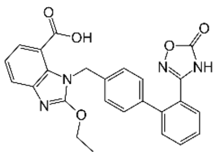

azilsartan
MW = 456.5 Da
inverse agonist
for AT1R

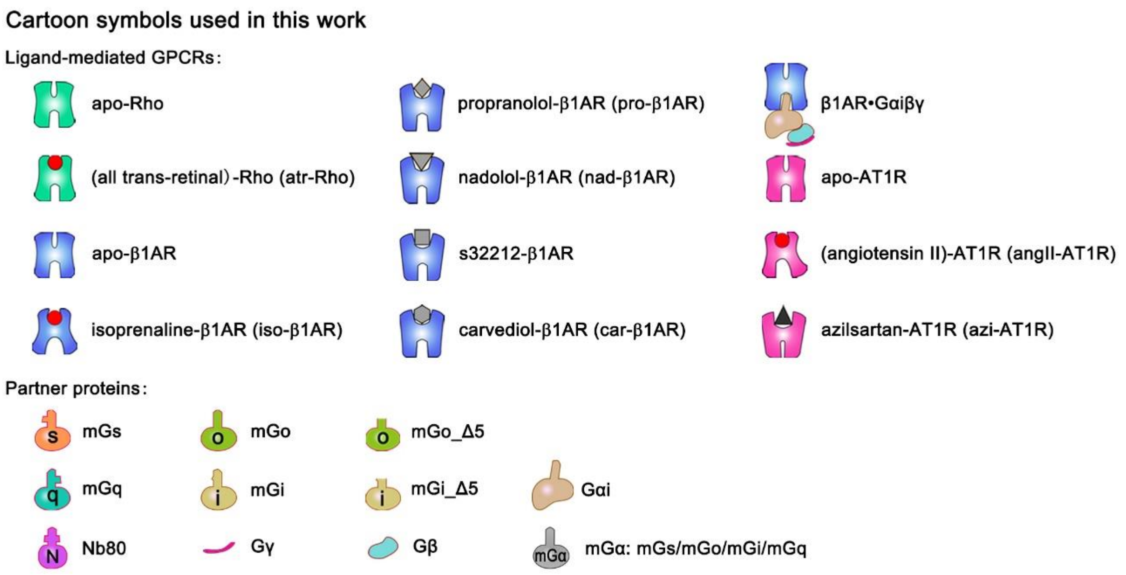

**Supplementary Table 4 |** Sequences of the GPCRs and G protein constructs used in this study

**Rho**

```
        10         20         30         40         50         60
MCGTEGPNFY VPFSNKTGVV RSPFEAPQYY LAEPWQFSML AAYMFLLIML GFPINFLTLY

        70         80         90        100        110        120
VTVQHKKLRT PLNYILLNLA VADLFMVFGG FTTTLYTSLH GYFVFGPTGC NLEGFFATLG

       130        140        150        160        170        180
GEIALWSLVV LAIERYVVVC KPMSNFRFGE NHAIMGVAFT WVMALACAAP PLVGWSRYIP

       190        200        210        220        230        240
EGMQCSCGID YYTPHEETNN ESFVIYMFVV HFIIPLIVIF FCYGQLVFTV KEAAAQQQES

       250        260        270        280        290        300
ATTQKAEKEV TRMVIIYVIA FLICWLPYAG VAFYIFTHQG SCFGPIFMTI PAFFAKTSAV

       310        320        330        340
YNPVIYIMMN KQFRNCMVTT LCCGKNPLGD DEASTTVSKT ETSQVAPA
```

**β1AR**

```
        10         20         30         40         50         60
MGAELLSQQW EAGMSLLMAL VVLLIVAGNV LVIAAIGSTQ RLQTLTNLFI TSLACADLVV

        70         80         90        100        110        120
GLLVVPFGAT LVVRGTWLWG SFLCELWTSL DVLCVTASVE TLCVIAIDRY LAITSPFRYQ

       130        140        150        160        170        180
SLMTRARAKV IICTVWAISA LVSFLPIMMH WWRDEDPQAL KCYQDPGCCE FVTNRAYAIA

       190        200        210        220        230        240
SSIISFYIPL LIMIFVYLRV YREAKEQIRK IDRASKRKTS RVMLMREHKA LKTLGIIMGV

       250        260        270        280        290        300
FTLCWLPFFL VNIVNVFNRD LVPKWLFVAF NWLGYANSAM NPIIYCRSPD FRKAFKRLLA

       310
FPRKADRRLH GSGLEVLFQ
```

**AT1R**

```
        10         20           30       40         50         60
MALNSSTEDG IKRIQDDCPR AGRHSYIFVM IPTLYSIIFV VGIFGNSLVV IVIYFYMKLK

        70         80         90        100        110        120
TVASVFLLNL ALADLCFLLT LPLWAVYTAM EYRWPFGNHL CKIASASVSF NLYASVWLLT

       130        140        150        160        170        180
CLSIDRYLAI VHPMKSRLRR TMLVAKVTCI IIWLMAGLAS LPAVIHRNVY FIENTNITVC

       190        200        210        220        230        240
AFHYESRNST LPIGLGLTKN ILGFLFPFLI ILTSYTLIWK ALKKAYEIQK NKPRNDDIFR

       250        260        270        280        290        300
IIMAIVLFFF FSWVPHQIFT FLDVLIQLGV IHDCKIADIV DTAMPITICI AYFNNCLNPL

       310        320        330
FYGFLGKKFK KYFLQ LLKYI PPKAVDAGTE TSQVAPA
```

**mGs**

```
       10         20         30         40         50         60
GIEKQLQKDK QVYRATHRLL LLGADNSGKS TIVKQMRILH GGSGGSGGTS GIFETKFQVD

       70         80         90        100        110        120
KVNFHMFDVG GQRDERRKWI QCFNDVTAII FVVDSSDYNR LQEALNDFKS IWNNRWLRTI

      130        140        150        160        170        180
SVILFLNKQD LLAEKVLAGK SKIEDYFPEF ARYTTPEDAT PEPGEDPRVT RAKYFIRDEF

      190        200        210        220
LRISTASGDG RHYCYPHFTC AVDTENARRI FNDCRDIIQR MHLRQYELL
```

**mGo**

```
       10         20         30         40         50         60
GIEKNLKEDG ISAAKDVKLL LLGADNSGKS TIVKQMKIIH GGSGGSGGTT GIVETHFTFK

       70         80         90        100        110        120
NLHFRLFDVG GQRSERKKWI HCFEDVTAII FCVDLSDYNR MHESLMDFDS ICNNKFFIDT

      130        140        150        160        170        180
SIILFLNKKD LFGEKIKKSP LTICFPEYTG PNTYEDAAAY IQAQFESKNR SPNKEIYCHM

      190        200        210
TCATDTNNAQ VIFDAVTDII IANNLRGCGL Y
```

**mGo_Δ5**

```
       10         20         30         40         50         60
GIEKNLKEDG ISAAKDVKLL LLGADNSGKS TIVKQMKIIH GGSGGSGGTT GIVETHFTFK

       70         80         90        100        110        120
NLHFRLFDVG GQRSERKKWI HCFEDVTAII FCVDLSDYNR MHESLMDFDS ICNNKFFIDT

      130        140        150        160        170        180
SIILFLNKKD LFGEKIKKSP LTICFPEYTG PNTYEDAAAY IQAQFESKNR SPNKEIYCHM

      190        200
TCATDTNNAQ VIFDAVTDII IANNLR
```

**mGi**

```
       10         20         30         40         50         60
GIEKQLQKDK QVYRATHRLL LLGADNSGKS TIVKQMRILH GGSGGSGGTS GIFETKFQVD

       70         80         90        100        110        120
KVNFHMFDVG GQRDERRKWI QCFNDVTAII FVVDSSDYNR LQEALNDFKS IWNNRWLRTI

      130        140        150        160        170        180
SVILFLNKQD LLAEKVLAGK SKIEDYFPEF ARYTTPEDAT PEPGEDPRVT RAKYFIRDEF

      190        200        210        220
LRISTASGDG RHYCYPHFTC AVDTENARRI FNDVTDIIIK MNLRDCGLF
```

**mGi_Δ5**

```
        10         20         30         40         50         60
GIEKQLQKDK QVYRATHRLL LLGADNSGKS TIVKQMRILH GGSGGSGGTS GIFETKFQVD

        70         80         90        100        110        120
KVNFHMFDVG GQRDERRKWI QCFNDVTAII FVVDSSDYNR LQEALNDFKS IWNNRWLRTI

       130        140        150        160        170        180
SVILFLNKQD LLAEKVLAGK SKIEDYFPEF ARYTTPEDAT PEPGEDPRVT RAKYFIRDEF

       190        200        210        220
LRISTASGDG RHYCYPHFTC AVDTENARRI FNDVTDIIIK MNLR
```

**mGq**

```
        10         20         30         40         50         60
GIEKQLQKDK QVYRATHRLL LLGADNSGKS TIVKQMRILH GGSGGSGGTS GIFETKFQVD

        70         80         90        100        110        120
KVNFHMFDVG GQRDERRKWI QCFNDVTAII FVVDSSDYNR LQEALNDFKS IWNNRWLRTI

       130        140        150        160        170        180
SVILFLNKQD LLAEKVLAGK SKIEDYFPEF ARYTTPEDAT PEPGEDPRVT RAKYFIRDEF

       190        200        210        220
LRISTASGDG RHYCYPHFTC AVDTENARRI FNDCKDIILQ MNLREYNLV
```

**Nb80**

```
        10         20         30         40         50         60
QVQLQESGGG LVQAGGSLRL SCAASGSIFS INTMGWYRQA PGKQRELVAA IHSGGSTNYA

        70         80         90        100        110        120
NSVKGRFTIS RDNAANTVYL QMNSLKPEDT AVYYCNVKDY GAVLYEYDYW GQGTQVTVSS

HHHHHH
```

**Gαi**

```
        10         20         30         40         50         60
GGSMGCTLSA EDKAAVERSK MIDRNLREDG EKAAREVKLL LLGAGESGKS TIVKQMKIIH

        70         80         90        100        110        120
EAGYSEEECK QYKAVVYSNT IQSIIAIIRA MGRLKIDFGD SARADDARQL FVLAGAAEEG

       130        140        150        160        170        180
FMTAELAGVI KRLWKDSGVQ ACFNRSREYQ LNDSAAYYLN DLDRIAQPNY IPTQQDVLRT

       190        200        210        220        230        240
RVKTTGIVET HFTFKDLHFK MFDVGGQRSE RKKWIHCFEG VTAIIFCVAL SDYDLVLAED

       250        260        270        280        290        300
EEMNRMHESM KLFDSICNNK WFTDTSIILF LNKKDLFEEK IKKSPLTICY PEYAGSNTYE

       310        320        330        340        350
EAAAYIQCQF EDLNKRKDTK EIYTHFTCAT DTKNVQFVFD AVTDVIIKNN LKDCGLF
```

**Gβ**

```
       10         20         30         40         50         60
MSELDQLRQE AEQLKNQIRD ARKACADATL SQITNNIDPV GRIQMRTRRT LRGHLAKIYA

       70         80         90        100        110        120
MHWGTDSRLL VSASQDGKLI IWDSYTTNKV HAIPLRSSWV MTCAYAPSGN YVACGGLDNI

      130        140        150        160        170        180
CSIYNLKTRE GNVRVSRELA GHTGYLSCCR FLDDNQIVTS SGDTTCALWD IETGQQTTTF

      190        200        210        220        230        240
TGHTGDVMSL SLAPDTRLFV SGACDASAKL WDVREGMCRQ TFTGHESDIN AICFFPNGNA

      250        260        270        280        290        300
FATGSDDATC RLFDLRADQE LMTYSHDNII CGITSVSFSK SGRLLLAGYD DFNCNVWDAL

      310        320        330        340
KADRAGVLAG HDNRVSCLGV TDDGMAVATG SWDSFLKIWN
```

**Gγ**

```
       10         20         30         40         50         60
MPVINIEDLT EKDKLKMEVD QLKKEVTLER MLVSKCCEEF RDYVEERSGE DPLVKGIPED

       70
KNPFKELKGG CVIS
```

**Supplementary Table 5 |** Association constants ($K_a = 1/K_d$) of the GPCR•partner protein complexes

| Complex | $K_a$ (μM$^{-1}$) | Complex | $K_a$ (μM$^{-1}$) | Complex | $K_a$ (μM$^{-1}$) |
|---|---|---|---|---|---|
| apo-Rho•mGo | 0.56±0.02 | iso-β1AR•mGs | 2.86±0.33 | apo-AT1R•mGo | 0.27±0.01 |
| apo-Rho•mGi | 1.01±0.02 | iso-β1AR•mGo | 4±0.32 | angII-AT1R•mGo | 1.02±0.07 |
| atr-Rho•mGo | 1.15±0.03 | iso-β1AR•mGi | 0.62±0.03 | angII-AT1R•mGq | 0.48±0.05 |
| atr-Rho•mGi | 1.3±0.1 | iso-β1AR•mGq | 0.81±0.02 | apo-β1AR• mGo_Δ5 | 3.57±0.38 |
| apo-β1AR•mGo | 4±0.16 | iso-β1AR•Nb80 | 4.76±0.68 | iso-β1AR• mGo_Δ5 | 4.17±0.52 |

**Supplementary Table 6 |** Comparision of $K_d$ values from MS method and other biophysical methods

| **GPCR•partner protein** | **$K_d$ (μM) (in paranthesis: detection method used)** | **$K_d$ (μM) measured via high-mass MALDI-MS)** |
|---|---|---|
| atr-Rho•mGo | 1.38±0.21 μM (MST; this work) | 0.87±0.02 |
| iso-β1AR•mGs | 0.2±0.001 (fluorescence-based saturation binding analysis) (10)<br>0.32±0.019 (fluorescence anisotropy–based affinity assay) (2) | 0.35±0.04 |
| iso-β1AR•Nb80 | 0.35±0.08 (isothermal calorimetry) (11)<br>0.33±0.03 ( fluorescence anisotropy–based affinity assay) (2) | 0.21±0.03 |

## SI REFERENCES


1. P. J. Reeves, N. Callewaert, R. Contreras, H. G. Khorana, Structure and function in rhodopsin: high-level expression of rhodopsin with restricted and homogeneous N-glycosylation by a tetracycline-inducible N-acetylglucosaminyltransferase I-negative HEK293S stable mammalian cell line. *Proc. Natl. Acad. Sci. U.S.A.* **99**, 13419–13424 (2002).

2. A. F. M. Gavriilidou, *et al.*, Insights into the Basal Activity and Activation Mechanism of the β1 Adrenergic Receptor Using Native Mass Spectrometry. *J. Am. Soc. Mass Spectrom.* **30**, 529–537 (2019).

3. S. Maeda, *et al.*, Crystallization Scale Preparation of a Stable GPCR Signaling Complex between Constitutively Active Rhodopsin and G-Protein. *PLoS ONE* **9**, e98714 (2014).

4. B. Carpenter, C. G. Tate, Engineering a minimal G protein to facilitate crystallisation of G protein-coupled receptors in their active conformation. *Protein Eng. Des. Sel.* **29**, 583–594 (2016).

5. S. G. F. Rasmussen, *et al.*, Structure of a nanobody-stabilized active state of the β2 adrenoceptor. *Nature* **469**, 175–180 (2011).

6. D. Sun, *et al.*, Probing Gαi1 protein activation at single-amino acid resolution. *Nat. Struct. Mol. Biol.* **22**, 686–694 (2015).

7. D. Mayer, *et al.*, Distinct G protein-coupled receptor phosphorylation motifs modulate arrestin affinity and activation and global conformation. *Nat. Commun.* **10**, 1261 (2019).

8. A. Waterhouse, *et al.*, SWISS-MODEL: homology modelling of protein structures and complexes. *Nucleic Acids Res.* **46**, W296–W303 (2018).

9. J. F. Armstrong, *et al.*, The IUPHAR/BPS Guide to PHARMACOLOGY in 2020: extending immunopharmacology content and introducing the IUPHAR/MMV Guide to MALARIA PHARMACOLOGY. *Nucleic Acids Res.* **48**, D1006-D1021 (2020).

10. R. Nehmé, *et al.*, Mini-G proteins: Novel tools for studying GPCRs in their active conformation. *PLoS ONE* **12**, e0175642 (2017).

11. J.L. Miller-Gallacher, *et al.*, The 2.1 Å resolution structure of cyanopindolol-bound β1-adrenoceptor identifies an intramembrane $Na^+$ ion that stabilises the ligand-free receptor. *PLoS ONE* **9,** e92727 (2014).
